# Identification of novel proteins for lacunar stroke by integrating genome-wide association data and human brain proteomes

**DOI:** 10.1186/s12916-022-02408-y

**Published:** 2022-06-23

**Authors:** Chengcheng Zhang, Fengqin Qin, Xiaojing Li, Xiangdong Du, Tao Li

**Affiliations:** 1 Mental Health Center and Psychiatric Laboratory, the State Key Laboratory of Biotherapy, West China Hospital of Sichuan University, Chengdu, Sichuan China; 2 Department of Neurology, the 3rd Affiliated Hospital of Chengdu Medical College, Chengdu, Sichuan China; 3 Suzhou Psychiatric Hospital, The Affiliated Guangji Hospital of Soochow University, Suzhou, Jiangsu China; 4 Department of Neurobiology, Affiliated Mental Health Center & Hangzhou Seventh People’s Hospital, Zhejiang University School of Medicine, Hangzhou Zhejiang, China; 5 NHC and CAMS Key Laboratory of Medical Neurobiology, MOE Frontier Science Center for Brain Science and Brain-machine Integration, School of Brain Science and Brain Medicine, Zhejiang University, Zhejiang, Hangzhou China

**Keywords:** Lacunar stroke, Human brain proteomes, Functional genomics, ICA1L, CAND2, ALDH2

## Abstract

**Background:**

Previous genome-wide association studies (GWAS) have identified numerous risk genes for lacunar stroke, but it is challenging to decipher how they confer risk for the disease. We employed an integrative analytical pipeline to efficiently transform genetic associations to identify novel proteins for lacunar stroke.

**Methods:**

We systematically integrated lacunar stroke genome-wide association study (GWAS) (*N*=7338) with human brain proteomes (*N*=376) to perform proteome-wide association studies (PWAS), Mendelian randomization (MR), and Bayesian colocalization. We also used an independent human brain proteomic dataset (*N*=152) to annotate the new genes.

**Results:**

We found that the protein abundance of seven genes (*ICA1L*, *CAND2*, *ALDH2*, *MADD*, *MRVI1*, *CSPG4*, and *PTPN11*) in the brain was associated with lacunar stroke. These seven genes were mainly expressed on the surface of glutamatergic neurons, GABAergic neurons, and astrocytes. Three genes (*ICA1L*, *CAND2*, *ALDH2*) were causal in lacunar stroke (*P* < 0.05/proteins identified for PWAS; posterior probability of hypothesis 4 ≥ 75 % for Bayesian colocalization), and they were linked with lacunar stroke in confirmatory PWAS and independent MR. We also found that *ICA1L* is related to lacunar stroke at the brain transcriptome level.

**Conclusions:**

Our present proteomic findings have identified *ICA1L*, *CAND2*, and *ALDH2* as compelling genes that may give key hints for future functional research and possible therapeutic targets for lacunar stroke.

**Supplementary Information:**

The online version contains supplementary material available at 10.1186/s12916-022-02408-y.

## Background

Lacunar stroke has been recognized as a stroke subtype for over 50 years, although the etiology and whether it differs from cortical ischemic stroke are still debated [1]. Approximately 30% of patients with lacunar stroke are left dependent, and up to 25% of patients are predicted to have another stroke within 5 years [2]. The increase in large-scale genome-wide association studies (GWAS) has greatly aided the discovery of genetic variations linked to lacunar stroke during the last decade [3]. However, deciphering the underlying biological processes responsible for the great majority of these genetic effects remains difficult, which has hampered the translation of these genetic results into novel drugs targeting these candidate genes for lacunar stroke [4].

Proteins are the most efficient biomarkers and therapeutic targets [5, 6] as they represent the major functional components of cellular and biological processes and the end products of gene expression [7]. It is critical to investigate the risk proteins in the brain disorders [8, 9]. Previous research on lacunar stroke examined genetic, epigenetic, and transcriptome variables [10, 11], but few studies have explored brain proteins directly [12]. For example, previous studies identified an association between loci on chromosome 16q24.2 and small vessel stroke in 4203 cases and 50,728 controls [13]. In addition, a transcriptome-wide association study identified associations between the expression of six genes (*SCL25A44*, *ULK4*, *CARF*, *FAM117B*, *ICA1L*, *NBEAL1*) and lacunar stroke [14]. The current breakthrough in high-throughput proteome sequencing of complex tissues [15, 16] represents a significant step forward in the large-scale quantification of the human brain proteome. Wingo et al. developed a novel framework called proteome-wide association studies (PWAS) to combine gene and protein expression data with the results of GWAS (integrate gene expression data and GWAS results) in depression pathogenesis [12]. Ou and colleagues also revealed that particular genetic variants impact disorders by altering the quantity of brain proteins, and uncovered potentially brain-pathogenic proteins in Alzheimer’s disease [17]. Thus, the causal inference of this integrated analytical approach has been empirically verified and shown to be reliable [17, 18].

Accordingly, we sought to discover novel drug targets for lacunar stroke by combining high-throughput proteomics in the brain with genetic data to determine the genomic architecture-associated protein levels. To identify potential protein biomarkers, we systematically linked protein biomarkers to lacunar stroke by taking a four-step approach. First, we used two protein quantitative trait locus (pQTL) datasets obtained from brain tissue and findings from lacunar stroke GWAS to perform a PWAS analysis. Second, we used independent Mendelian randomization (MR) analysis to verify PWAS-significant genes. Third, we used a COLOC to integrate GWAS data and brain pQTL using a Bayesian colocalization analysis to explore whether two associated signals are consistent with shared causal variant(s). Fourth, we explored the significant genes driving GWAS signals at the transcriptional level by leveraging gene expression data.

## Methods and materials

### Human brain protein abundance references in the discovery PWAS

The discovery PWAS data were obtained from the dorsolateral prefrontal cortex (dPFC) of postmortem brain tissues from 376 subjects recruited by the Religious Orders Study/Memory and Aging Project (ROS/MAP) [19]. For proteome sequencing, isobaric tandem mass tag peptide labeling was utilized, and peptides were assessed using liquid chromatography coupled to mass spectrometry (MS) [20]. Wingo et al. [21] used Thermo Fisher Scientific’s Proteome Discoverer suite v.2.3 and tandem MS spectra to search against the standard UniProtKB human proteome database, which has 20,338 total sequences, to assign peptide spectral matches. Genotyping was done using whole-genome sequencing or genome-wide genotyping on the Illumina OmniQuad Express or Affymetrix GeneChip 6.0 platforms. Over 8356 proteins having both proteomic and genomic data, among which 1475 protein could find significant cis associations with genetic variation.

### Human brain protein abundance references in the confirmation PWAS

The confirmation PWAS data were profiled from the dPFC of postmortem brain samples from 198 participants recruited by the Banner Sun Health Research Institute (Banner) [22]. Proteomic profiling followed the same steps as the discovery proteomes, with two exceptions: only MS2 scans were collected, and MS2 spectra were compared to the UniProtKB human brain proteome database [22]. Individuals from Banner were genotyped using an Affymetrix Precision Medicine Array following the manufacturer’s protocol and DNA extracted from the brain with a Qiagen GenePure kit [20]. Following quality control [12], we included 152 individuals having pQTL data in our replication analysis.

### Human brain eQTL in the lacunar stroke TWAS

Transcriptomes data were profiled from postmortem brain samples donated by 452 individuals recruited by the CommonMind Consortium (CMC) [23]. These transcriptomes were profiled mainly from the dPFC. The RNA-seq data were adjusted for diagnosis, institution collecting the data, sex, disease onset age, postmortem interval (PMI), RNA integrity number (RIN), RIN^2^, clustered library batch variable, and 20 surrogate variables. The eQTL was calculated according to the formula: adjusted gene expression ~ SNP dosage + ancestry vectors + diagnosis. We retrieved the gene-level eQTL results adjusted with surrogate variable analysis. Detailed information can be found in the original study [23].

### Lacunar stroke GWAS data

We used the summary association statistics from the largest GWAS of lacunar stroke by Traylor et al. [14], which included 7338 cases and 254,798 controls of European, South Asian, African, and Hispanic ancestry recruited from hospitals across the UK as part of the UK DNA Lacunar Stroke studies 1 and 2 and the International Stroke Genetics Consortium. The current study used lacunar stroke samples mostly from the magnetic resonance imaging (MRI) verified and traditional phenotypic groups. In the MRI-confirmed group, lacunar stroke was defined as a clinical lacunar syndrome with an anatomically compatible lesion on MRI, either as (i) a high intensity region on diffusion-weighted imaging for acute infarcts or (ii) a low intensity region on fluid-attenuated inversion recovery or (iii) T1 imaging for non-acute infarcts, and the absence of other causes of stroke other than small vessel disease. In the traditional phenotyping group, lacunar stroke was also classified using the TOAST criteria, which is comprised of a clinical lacunar syndrome and the absence of other types of stroke, as well as non-lacunar infarction on CT. MRI identified 2987 patients with lacunar stroke accounting for 40.7% of all lacunar stroke patients. Meta-analysis was performed as previously described [24] by METAL tool using the fixed-effects inverse-variance weighted model [25]. Following meta-analysis, the λ_1000_ value in the transethnic analysis covering European, South Asian, African, and Hispanic was 1.005, showing no significant inflation [14]. Detailed information about the study subjects, diagnosis, genotyping, quality control, and statistical analyses was provided in the original papers [14].

### Statistical analysis

#### Proteome-wide association studies (PWAS)

PWAS were carried out using FUSION [26]. For simplicity, we used FUSION to compute the effect of SNPs on protein abundance for proteins with significant heritability (heritability *P* < 0.01). Multiple predictive models, top1, blup, lasso, enet, and bslmm, were adopted in the analysis [26]. Protein weights from the most predictive model were selected. Subsequently, we used FUSION to combine the genetic effect of lacunar stroke (lacunar stroke GWAS *z*-score) with the protein weights by calculating the linear sum of *z-*score × weight for the independent SNPs at the locus to perform the PWAS of lacunar stroke.

#### Mendelian Randomization (MR) analysis

MR was used to verify whether lacunar stroke PWAS-significant genes (from the FUSION approach) were associated with lacunar stroke via their cis-regulated brain protein abundance. The SNPs included in the study robustly and independently (*R*^2^ < 0.001) predicted exposures at a genome-wide level (5 × 10^−8^). The Wald ratio calculates the log odds change in lacunar stroke risk per standard deviation change in protein biomarker in relation to the instrumenting SNP’s risk allele [27]. A weighted mean of the ratio estimates weighted by the inverse variance of the ratio estimates (inverse-variance weighted approach) was employed when more than one SNP was available [28]. Complementary approaches were also used, such as weighted median, MR-Egger, simple mode, and weighted mode. To construct MR estimates, the “TwoSampleMR” package [29] in R 4.1.02 was utilized.

#### Bayesian colocalization analysis

To assess the probability of the same single-nucleotide variation being responsible for both changing the lacunar stroke risk and modulating the protein levels of a gene, we used the COLOC method [26, 30]. We used the default COLOC priors of p1 = 10^−4^, p2 = 10^−4^, and p12 = 10^−5^, where p1 is the probability that a given variant is associated with lacunar stroke, p2 is the probability that a given variant is a significant pQTL, and p12 is the probability that a given variant is both a lacunar stroke result and an pQTL. COLOC uses computed approximation Bayes factors and summary association data to generate posterior probability for the following 5 hypotheses: H_0_, No association with either GWAS or pQTL; H_1_, Association with GWAS, not with pQTL; H_2_, Association with pQTL, not with GWAS; H_3_, Association with GWAS and pQTL, two independent SNPs; and H_4_, Association with GWAS and pQTL, one shared SNP. The posterior probability (PP), represented by PP0, PP1, PP2, PP3, and PP4, quantifies support for each of the hypotheses. H_4_ of 0.75 or above were chosen as strong evidence for colocalization.

#### Transcriptome-wide association studies (TWAS)

Using FUSION [26], which generates the linear sum of *Z* score weights for the independent SNPs at the locus, then the genetic influence of lacunar stroke (lacunar stroke GWAS *Z* score) was combined with the mRNA expression weights. The following was the fundamental procedure: firstly, FUSION computed TWAS expression weights (i.e., SNP-gene expression correlations) from the reference expression panels (i.e., CMC) [23]. To identify the best gene prediction model, FUSION did a fivefold cross-validation of each model to obtain an out-sample *R*^2^ [26]. The imputed gene expression was then used to investigate the association with lacunar stroke [31].

#### Cell-type specificity analysis

Using human brain single-cell RNA sequencing (RNA-seq) data profiled from the Cell Types database (https://portal.brain-map.org/atlases-and-data/rnaseq), we investigated the cell type-specific expression of the risk genes. Individual layers of the cortex were dissected, and nuclei were dissociated and sorted using the neuronal marker NeuN from human brain tissues. The expression was profiled with SMART-Seq v4 or 10× Genomics Chromium Single Cell 3’ v3 RNA-seq. CELLEX (CELL-type EXpression-specificity), a method for generating cell-type expression specificity (ES) profiles, was used to obtain gene expression specificity values [32, 33].

## Results

### Discovery and replication PWAS of lacunar stroke

**Fig. 1:**
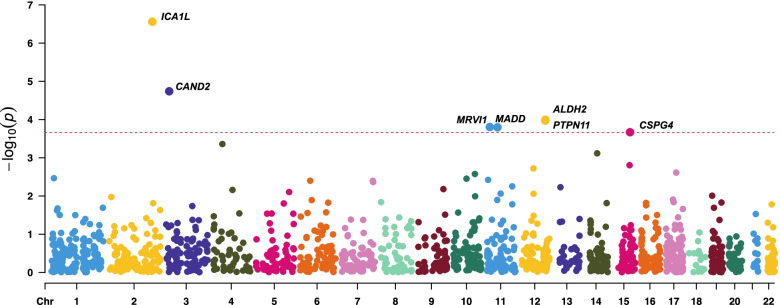
Manhattan plot for the discovery lacunar stroke PWAS integrating the lacunar stroke GWAS (*N* = 7338) with the discovery ROS/MAP proteomes (*N* = 376). Each point represents a single association test between a gene and lacunar stroke ordered by genomic position on the *x* axis and the association strength on the *y* axis as the −log_10_(*P*) of a *z*-score test. The discovery PWAS identified 7 genes whose *cis*-regulated brain protein abundance was associated with lacunar stroke at an FDR of *P* < 0.05. The red horizontal line reflects the significant threshold of the FDR *P* < 0.05 and is set at the highest unadjusted *P* value that is below that threshold (*P* = 2.2 × 10^−4^)

The PWAS identified 7 genes (*ICA1L*, *CAND2*, *ALDH2*, *MADD*, *MRVI1*, *CSPG4*, and *PTPN11*) whose cis-regulated brain protein levels were associated with lacunar stroke at a false discovery rate (FDR) of *P*<0.05 (Additional file 1: Table S1). Four genes (*ICA1L*, *CAND2*, *ALDH2*, and *MADD*) could be replicated in the independent PWAS of lacunar stroke, providing a higher confidence level (Fig. 1 and Table 1). Three of the 7 significant proteins from the discovery PWAS could not be tested in the confirmation PWAS, with 2 proteins (CSPG4 and PTPN11) not profiled, and MRVI1 was profiled but did not have substantial heritability, which is likely due to the smaller sample size (Table 1).

**Table 1:** The discovery lacunar stroke PWAS identified 7 significant genes, of which 5 were found in the confirmation PWAS, and all 4 replicated

			Discovery PWAS			Confirmation PWAS		Evidence for replication
	Gene	Chromosome	PWAS ***z-***score	PWAS ***P***	PWAS FDR ***P***	PWAS ***z***-score	PWAS ***P***	
1	CAND2	3	−4.281	1.86E−05	1.36E−02	−4.334	1.46E−05	Yes
2	ICA1L	2	−5.136	2.81E−07	4.11E−04	−4.213	2.52E−05	Yes
3	ALDH2	12	3.871	1.09E−04	3.98E−02	3.567	3.61E−04	Yes
4	MADD	11	3.770	1.63E−04	3.98E−02	2.917	3.53E−03	Yes
5	MRVI1	11	−3.776	1.60E−04	3.98E−02	−1.680	9.30E−02	-
6	CSPG4^a^	15	3.696	2.19E−04	4.58E−02	-	-	
7	PTPN11^a^	12	−3.884	1.03E−04	3.98E−02	-	-	

This table gives the *z*-scores for the lacunar stroke PWAS associations with their corresponding *P* values and FDR-adjusted *P* values for all significant genes in the lacunar stroke discovery PWAS. Confirmation lacunar stroke PWAS *z*-scores and their corresponding unadjusted *P* values are provided for the significant genes in the discovery lacunar stroke PWAS

^a^Protein not profiled in the confirmation proteomic dataset

### Cell-type specificity analysis in the brain

**Fig. 2:**
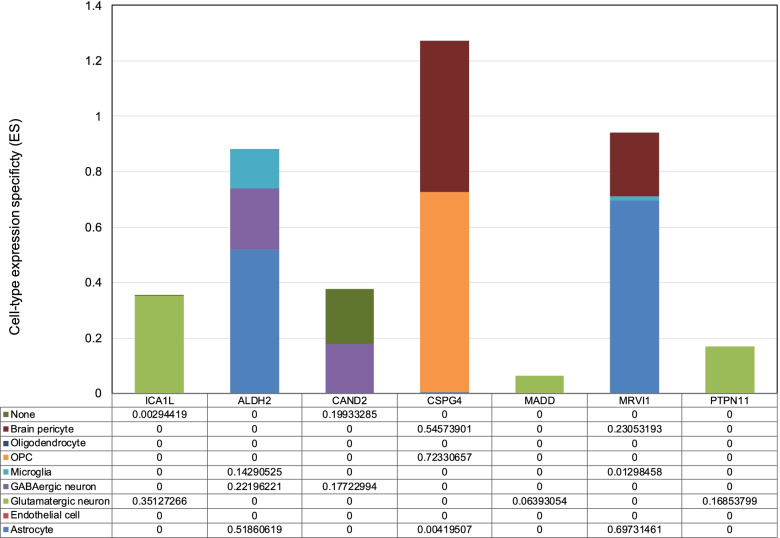
Single-cell-type expression of the potentially lacunar stroke-risk genes. Bar graph of single-cell-type enrichment for risk genes in lacunar stroke from the discovery PWAS. The diagram depicts CELL-type EXpression-specificity (*y* axis) for each gene (*x* axis), with evidence of substantial enrichment within a specific brain cell type (histogram of the bar). We used the “wisdom of the crowd” technique to assess enrichment based on gene expression in one cell type against all other cell types. OPC, oligodendrocyte precursor cell. None: Cell types that cannot be classified

We investigated whether the risk genes identified by PWAS were enriched in a particular brain cell type. Using human single-cell RNA-seq data from the Cell Types database (https://portal.brain-map.org/atlases-and-data/rnaseq), we found cell type-specific enrichment for the expression of the seven causal genes (Fig. 2). *MRVI1* and *ALDH2* were found to be more abundant in astrocytes, whereas *ICA1L*, *PTPN11*, and *MADD* were only found in glutamatergic neurons. GABAergic neurons had higher levels of *CAND2* and *ALDH2*.

### MR verify 4 genes associated with lacunar stroke using brain pQTL

Most of the analyzed proteins could only be instrumented using a single SNP; thus, MR estimates were mainly based on the Wald ratio method. We further confirmed four proteins, including ICA1L, CAND2, ALDH2, and MADD, biomarkers that revealed significant evidence of a connection in the lacunar stroke GWAS (Table 2).

**Table 2:** Risk genes verified by Mendelian randomization (MR) and colocalization using brain pQTL

	Gene	Beta	SE	***P*** value	Evidence for replication	H_**4**_	PP4/(PP3+PP4)	Causal variant
1	CAND2	−0.758	0.176	1.71E−05	Yes	0.94	0.99	Yes
2	ICA1L	−2.531	0.493	2.81E−07	Yes	0.99	0.99	Yes
3	ALDH2	1.157	0.314	2.28E−04	Yes	0.75	0.92	Yes
4	MADD	1.161	0.398	3.53E−03	Yes	-	-	-

This table shows the Beta, SE, and *P* values for the MR. MR *P* values and a direction of effect consistent with the discovery and replication PWAS results. For the 4 FDR-significant genes in the discovery lacunar stroke PWAS, the result of COLOC H_4_, which is the Bayesian posterior probability that a genetic variant is shared by both traits (that is, the genetically regulated protein level and lacunar stroke)

### Colocalization between lacunar stroke risk genes and pQTLs in the brain

Lacunar stroke PWAS associations may arise from a coincidental overlap between pQTLs and sites in linkage disequilibrium with lacunar stroke GWAS sites or from a variant associated with protein expression (the variant is a protein quantitative trait locus (pQTL)) and lacunar stroke at the same time. Statistical colocalization analysis reported for each gene, the probability that the GWAS and pQTL share a causal variant, referred to as both hypothesis 4 (H_4_) and PP4/(PP3+PP4) ≥ 0.75. Based on a H_4_ ≥75 percent and PP4/(PP3+PP4) ≥ 0.75, this analysis revealed three of the seven genes (ICA1L, CAND2, and ALDH2) that offered evidence of genetic colocalization (Table 2). It suggests that these three proteins play an important role in the pathophysiology of lacunar stroke.

### Specificity of the lacunar stroke PWAS results

**Fig. 3:**
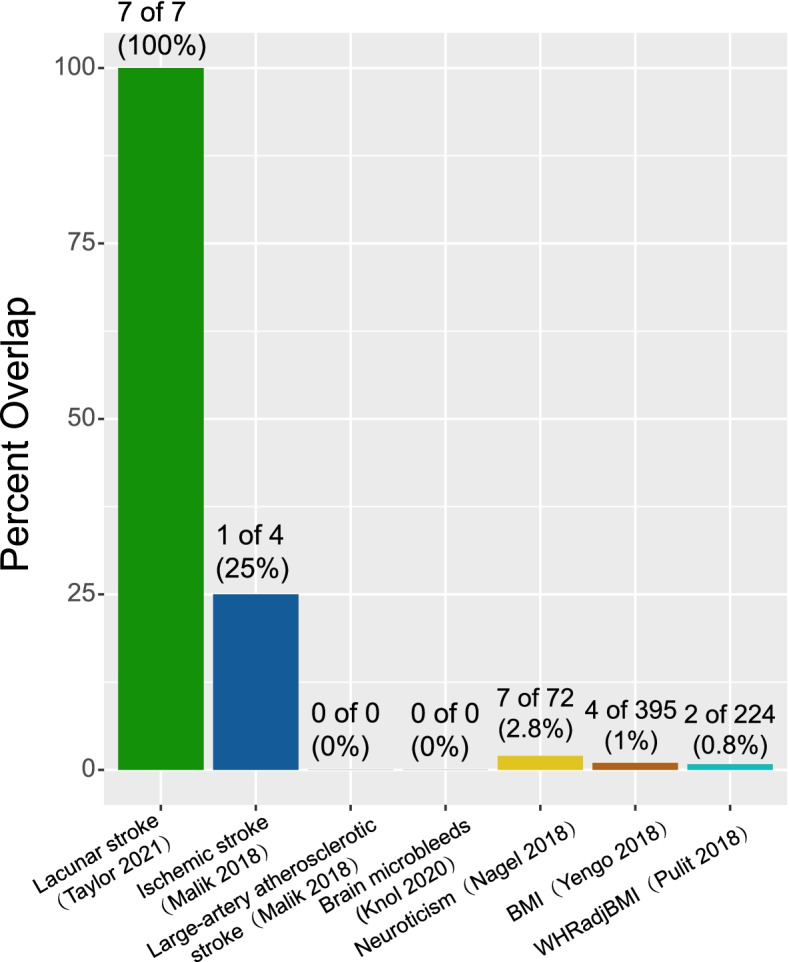
Overlap of significant genes between lacunar stroke and other traits. Overlap between results of the lacunar stroke PWAS and PWAS for other traits. The PWAS used the discovery ROS/MAP proteomic dataset (*N* = 376) and GWAS summary results. The following outcomes were tested: ischemic stroke (*N* =60,341), large-artery atherosclerotic stroke (*N* = 6688), brain microbleeds (*N* = 3556), neuroticism (*N* = 390,278), body mass index (BMI; *N* = 681,275), and waist-to-hip ratio adjusting for BMI (WHRadjBMI; *N* = 694,649). Significant genes considered for overlap are those with FDR *P* < 0.05

We did PWAS for other brain-related and biologic traits to understand the specificity of PWAS results for lacunar stroke, and we predicted the degree of overlap of important genes to roughly correlate to their genetic relationship. GWAS results from ischemic stroke (*N* =60,341) [34], large-artery atherosclerotic stroke (*N* = 6688) [34], brain microbleeds (*N* = 3556) [35], neuroticism (*N* = 390,278) [36], body mass index (BMI; *N* = 681,275) [37], and waist-to-hip ratio adjusting for BMI (*N* = 694,649) [38] were combined with the discovery proteomic profiles to perform PWAS of each trait. Using FUSION, the PWAS of ischemic stroke identified 4 genes, while the PWAS of large-artery atherosclerotic stroke and brain microbleeds identified none. The PWAS of neuroticism, BMI, and WHRadjBMI, as reported by Wingo and colleagues [37], identified 72, 395, and 244 genes, respectively (FDR *P* < 0.05) (Additional file 1: Table S2-7). As expected, the lacunar stroke PWAS found that 1 in 4 (ALDH2; 25%) ischemic stroke genes overlapped with 7 lacunar stroke PWAS-significant genes, reflecting their high degree of genetic correlation. Two of 72 (2.8%) neuroticism genes (MADD and ICA1L), 4 of 395 (1%) BMI genes (MADD, ICA1L, CSPG4, and PTPN11), and 2 of 244 (0.8%) WHRadjBMI genes (CAND2 and CSPG4) overlapped with 7 lacunar stroke PWAS-significant genes. There were no overlapping genes between large-artery atherosclerotic stroke, brain microbleeds, and lacunar stroke (Fig. 3).

### Examination of the potential lacunar stroke-causal proteins at the mRNA level

We combined the lacunar stroke GWAS data with human brain transcriptomes to conduct a lacunar stroke transcriptome-wide association analysis (TWAS) using FUSION. We found that the cis-regulated brain mRNA expression of the seven genes was associated with lacunar stroke (FDR *P *< 0.05) (Additional file 1: Table S8). Interestingly, we found one of the seven genes (*ICA1L*; Table 3; Additional file 1: Table S9) identified in the discovery PWAS, suggesting joint evidence from PWAS and TWAS for its role in lacunar stroke etiology.

**Table 3:** Summary of the 3 lacunar stroke PWAS-significant genes with evidence for being consistent with a causal role in lacunar stroke

	Gene	Chromosome	Discovery PWAS	Confirmation PWAS	Confirmation MR	Evidence for causality	TWAS significant
						COLOC	
1	ICA1L	2	Significant	Replicated	Replicated	Yes	Yes
2	CAND2^*^	3	Significant	Replicated	Replicated	Yes	N/A
3	ALDH2^*^	12	Significant	Replicated	Replicated	Yes	No

N/A, refers to genes that did not have significant heritability estimates to be included in the TWAS of lacunar stroke

*New gene refers to genes not within a 1-Mb window of SNPs with *P* < 5.23 × 10^−8^ identified in Taylor et al. lacunar stroke GWAS

### Significance of the protein findings

To determine the importance of the 7 potentially causal genes identified from the meta-analysis of the discovery and replication PWAS analyses, we obtained the lowest *P* values for the SNPs within 1 Mb of each of these 7 genes using the summary statistics from the most extensive lacunar stroke GWAS (*N* = 7338) [14]. The most significant *P* values were less than 5 × 10^–8^ in two genes (*MADD* and *ICA1L*), while the *P* values of SNPs in the remaining 5 genes ranged from 5.2×10^–5^ to 1.08×10^–7^ (Additional file 1: Table S10). The PWAS findings suggest that specific brain proteins likely contribute to the pathogenesis of lacunar stroke.

## Discussion

In the present study, we employed a pipeline of analytical techniques investigating the functional associations between protein biomarkers in the brain and lacunar stroke risk. We identified 7 potential risk genes (*ICA1L, CAND2, ALDH2, MADD, MRVI1, CSPG4*, and *PTPN11*) of lacunar stroke with altered protein abundances in the brain. Four (*ICA1L*, *CAND2*, *ALDH2*, *MADD)* of these 7 genes were replicated in the independent PWAS and MR validation analyses of lacunar stroke, providing a higher confidence level. Furthermore, we identified *ICA1L*, *CAND2*, and *ALDH2* from comprehensive analyses, including non-lacunar stroke brain PWAS and colocalization, and *ICA1L* was supported at the brain transcriptional level. These genes may serve as promising targets for further mechanistic and therapeutic studies.

Identifying therapeutic targets for diseases is a crucial goal of human genetics research and is particularly vital for neurovascular diseases, including lacunar stroke. Our analysis implicated genes previously investigated in lacunar stroke, such as *ICA1L* and *MADD*, as well as new candidates, including *CAND2*, *ALDH2*, *MRVI1*, *CSPG4*, and *PTPN11*. Two genes (*ICA1L* and *MADD*) reported in lacunar stroke play roles at the synapse. *ICA1L* encodes a protein triggered by type IV collagen and plays a crucial role in myelination [39]. According to our lacunar stroke PWAS data, ICA1L has a lower abundance in the brains of lacunar stroke patients. Furthermore, we discovered that ICA1L was enriched in cortical glutamate neurons. Glutamate neurons are crucial components in neural development and neuropathology through their role in cell proliferation, differentiation, survival, and neural network formation. Our findings imply that decreased *ICA1L* may impair excitatory synaptic signaling and contribute to the pathogenesis of lacunar stroke. *ICA1L* has also been linked to the etiology of lacunar stroke in previous transcriptome investigations [14]. Our findings show that *MADD* is more abundant in glutamate neurons. We speculate that *MADD* is primarily involved in the transmission of apoptotic signals in neuronal signaling pathways [40], consistent with previous research suggesting that ischemia causes excitatory glutamate toxicity [41–44].

Other notable molecular roles for the 5 novel genes in lacunar stroke include cerebral cavernous malformations, vascular inflammation, platelet adhesion, and cell apoptosis. *CAND2*, which encodes cullin-associated and neddylation-dissociated 2, plays a role in cerebral cavernous malformations [45]. Cavernous malformation is a key inducing factor in lacunar stroke and cerebral microbleeds [46]. According to our findings, *CAND2* is decreased, predominantly in GABAergic neurons in the brains of lacunar stroke patients, indicating its role in the etiology of lacunar stroke. Both *ALDH2* and *MRVI1* are involved in platelet adhesion [47] and vascular inflammation [48, 49]. Previous research has linked increased blood-brain barrier permeability to an inflammatory process involving activated monocytes/macrophages in individuals with cerebral small vessel disease [50, 51]. In our study, *MRVI1* was more abundant in astrocytes, which supports their roles in vascular inflammation. CSPG4, also known as neuron-glial antigen 2 (NG2) [52], is a protein that helps to stabilize cell-substrate connections [53–55]. Finally, we discovered a novel protein, PTPN11, as a new candidate for a membrane protein that suppresses cell growth and induces apoptosis [56–59]. These 7 genes are implicated in the molecular process and neuropathological changes in lacunar stroke.

Most trait-associated variants in neuropsychiatric disease are found in protein-noncoding areas of the human genome, where they have previously been linked to transcriptional levels [60–62]. As such, we applied eQTLs to understand GWAS-related transcriptional regulatory mechanisms in lacunar stroke. However, only the *ICA1L*-identified proteins exhibited changes in gene expression. There could be several reasons for this lack of agreement. First, while the exact link between eQTLs and pQTLs has yet to be discovered, the mRNA expression and protein levels of many genes are uncorrelated, owing in part to various posttranscriptional factors such as sequence characteristics implicated in protein translation and degradation [63]. Second, assay technical artifacts and differences in data analysis may impact the results significantly. While opposed to pQTL analysis [64], eQTL studies use stricter criteria to detect remote regulatory changes, resulting in a lower false-positive rate. In addition to raising thresholds, one way to improve the performance is to use strong tools like FUSION [26], MR [29], and COLOC [26, 30] to check findings with independent samples. To address this difficulty, however, it is essential to expand the depth and variety of multiomics sequencing at the individual level.

Clinical trials have been conducted using drug compounds targeting one of the three causal genes, including *ALDH2* (ranked as high confidence level in our findings), for alcohol dependency and parasite infection (two drugs, phase 4) [65]. Secondary analysis of those and future drugs in clinical trials would likely be helpful to prove the idea that the proteins are involved in the development of lacunar stroke.

Our study has several advantages. First, PWAS of lacunar stroke was conducted using the largest and most comprehensive human proteome and summary statistics from the most recent lacunar stroke GWAS. Second, we performed the replication PWAS using independent human brain proteome and verified the risk proteins with independent MR validation analysis. Third, based on Bayesian colocalization used to estimate the probability that two associated signals were observed at a particular site with a common causal variant, we confirmed the pathogenetic protein (ICA1L, CAND2, and ALDH2) of lacunar stroke. Fourth, this study analyzed both mRNA and protein levels associated with lacunar stroke utilizing both the PWAS and the TWAS. Finally, the dorsolateral prefrontal cortex in the current study was chosen because it includes the cell type most linked to lacunar stroke [14]. Furthermore, the prefrontal cortex has been proposed as a top-down control system that connects other brain areas to facilitate sophisticated cognitive functions. Prefrontal brain risk protein screening for lacunar stroke may help identify critical targets for enhanced cognitive function as well as those who are at high risk of stroke recurrence [2, 66].

The current study has several limitations. First, pQTL and eQTL mapping cannot solve all GWAS signals. At a single level, such as the protein level, the function of genes in the biological development of lacunar stroke is difficult to explain. More epigenetic investigations, based on mQTL, single-cell sequencing, and whole-genome sequencing, are needed to design tailored therapy regimens and offer a complete understanding of the molecular mechanisms implicated in lacunar stroke [67, 68]. Second, the method for detecting Slow Off-rate Modified Aptamers was limited to a subset of proteomes and did not cover the whole proteome. Third, because current proteome samples vary by ethnicity, further expansion of the scale and diversity of brain proteome data can help with more precise estimates and enable its broader applications.

## Conclusions

In conclusion, we found strong evidence supporting three novel brain proteins (ICA1L, CAND2, and ALDH2*)* associated with lacunar stroke. ICA1L was further verified at the mRNA level. These findings offer information on the genetic and physiological processes that underpin lacunar stroke, allowing novel therapeutic targets to be identified. Future research should take advantage of greater large-scale molecular datasets obtained from lacunar stroke-relevant tissues, which might provide unique insights into genetic and functional processes and identify potential druggable targets for new lacunar stroke treatments.

## Supplementary Information

**Additional file 1: Table S1.** The discovery lacunar stroke PWAS identified 7 significant genes. **Table S2.** The PWAS of ischemic stroke integrating the ischemic stroke GWAS (*N* = 60,341) with ROS/MAP human brain proteomic and genetic data (*N* = 376) using FUSION. **Table S3.** The PWAS of large-artery atherosclerotic stroke integrating the large-artery atherosclerotic stroke GWAS (*N* = 6,688) with ROS/MAP human brain proteomic and genetic data (*N* = 376) using FUSION. **Table S4.** The PWAS of brain microbleeds integrating the brain microbleeds GWAS (*N* = 3,556) with ROS/MAP human brain proteomic and genetic data (*N* = 376) using FUSION. **Table S5.** The PWAS of neuroticism integrating the neuroticism GWAS (*N* = 390,278) with ROS/MAP human brain proteomic and genetic data (*N* = 376) using FUSION. **Table S6.** The PWAS of BMI integrating the BMI GWAS (*N* = 681,275) with ROS/MAP human brain proteomic and genetic data (*N* = 376) using FUSION. **Table S7.** The PWAS of WHRadjBMI integrating the WHRadjBMI GWAS (*N* = 694,649) with ROS/MAP human brain proteomic and genetic data (*N* = 376) using FUSION. **Table S8.** The TWAS of lacunar stroke integrating the lacunar stroke GWAS (*N* = 7,338) with CMC human brain transcriptome and genetic data (*N* = 452) using FUSION. **Table S9.** The lacunar stroke TWAS verified 1 significant gene. **Table S10.** SNPs located within 1 Mb of each of the 7 proteins with the lowest *p*-value for association with lacunar stroke.

## Data Availability

All data relevant to the study are included in the article or uploaded as online supplementary information. The data generated in this study will be available from the corresponding author on reasonable request. GWAS summary statistics from these analyses are available at GWAS Catalog and on the Cerebrovascular Disease Knowledge Portal.

## Abbreviations

DLPFC: Dorsolateral prefrontal cortex
eQTL: Expression quantitative trait locus
GWAS: Genome-wide association studies
MR: Mendelian randomization
PGC: Psychiatric Genomics Consortium
pQTL: Protein quantitative trait locus
PWAS: Proteome-wide association studies
SNPs: Single-nucleotide polymorphisms
TWAS: Transcriptome-wide association study
