# Supplementary material for: Identification of novel proteins for lacunar stroke by integrating genome-wide association data and human brain proteomes
Source: BMC Med. 2022 Jun 23;20:211. doi: 10.1186/s12916-022-02408-y (PMC9219149; doi:10.1186/s12916-022-02408-y)
Supplement: Supplementary file 1 — Additional file 1: Table S1. The discovery lacunar stroke PWAS identified 7 significant genes. Table S2. The PWAS of ischemic stroke integrating the ischemic stroke GWAS (N = 60,341) with ROS/MAP human brain proteomic and genetic data (N = 376) using FUSION. Table S3. The PWAS of large-artery atherosclerotic stroke integrating the large-artery atherosclerotic stroke GWAS (N = 6,688) with ROS/MAP human brain proteomic and genetic data (N = 376) using FUSION. Table S4. The PWAS of brain microbleeds integrating the brain microbleeds GWAS (N = 3,556) with ROS/MAP human brain proteomic and genetic data (N = 376) using FUSION. Table S5. The PWAS of neuroticism integrating the neuroticism GWAS (N = 390,278) with ROS/MAP human brain proteomic and genetic data (N = 376) using FUSION. Table S6. The PWAS of BMI integrating the BMI GWAS (N = 681,275) with ROS/MAP human brain proteomic and genetic data (N = 376) using FUSION. Table S7. The PWAS of WHRadjBMI integrating the WHRadjBMI GWAS (N = 694,649) with ROS/MAP human brain proteomic and genetic data (N = 376) using FUSION. Table S8. The TWAS of lacunar stroke integrating the lacunar stroke GWAS (N = 7,338) with CMC human brain transcriptome and genetic data (N = 452) using FUSION. Table S9. The lacunar stroke TWAS verified 1 significant gene. Table S10. SNPs located within 1 Mb of each of the 7 proteins with the lowest p-value for association with lacunar stroke. [file 12916_2022_2408_MOESM1_ESM.docx]

**Supplementary material for**

**Identification of novel proteins for lacunar stroke by integrating genome-wide association data and human brain proteomes**

Chengcheng Zhang; Fengqin Qin; Xiaojing Li; Xiangdong Du; Tao Li

**Supplementary tables**

Table S1. The discovery lacunar stroke PWAS identified 7 significant genes.

Table S2. The PWAS of ischemic stroke integrating the ischemic stroke GWAS (N=60,341) with ROS/MAP human brain proteomic and genetic data (N=376) using FUSION.

Table S3. The PWAS of large-artery atherosclerotic stroke integrating the large-artery atherosclerotic stroke GWAS (N=6,688) with ROS/MAP human brain proteomic and genetic data (N=376) using FUSION.

Table S4. The PWAS of brain microbleeds integrating the brain microbleeds GWAS (N=3,556) with ROS/MAP human brain proteomic and genetic data (N=376) using FUSION.

Table S5. The PWAS of neuroticism integrating the neuroticism GWAS (N=390,278) with ROS/MAP human brain proteomic and genetic data (N=376) using FUSION.

Table S6. The PWAS of BMI integrating the BMI GWAS (N=681,275) with ROS/MAP human brain proteomic and genetic data (N=376) using FUSION.

Table S7. The PWAS of WHRadjBMI integrating the WHRadjBMI GWAS (N=694,649) with ROS/MAP human brain proteomic and genetic data (N=376) using FUSION.

Table S8. The TWAS of lacunar stroke integrating the lacunar stroke GWAS (N=7,338) with CMC human brain transcriptome and genetic data (N=452) using FUSION.

Table S9. The lacunar stroke TWAS verified 1 significant gene.

Table S10. SNPs located within 1 Mb of each of the 7 proteins with the lowest p-value for association with lacunar stroke.

| Table S1. The discovery lacunar stroke PWAS identified 7 significant genes. | | | | | |  |  |
| --- | --- | --- | --- | --- | --- | --- | --- |
| **Gene** | **CHR** | **pQTL.ID** | **pQTL.R^2^** | **NSNP** | **MODEL** | **MODELCV.R^2^** | **MODELCV.PV** |
| ICA1L | 2 | rs7582720 | 0.115769 | 81 | top1 | 0.12 | 7.30E-12 |
| CAND2 | 3 | rs4642101 | 0.329858 | 95 | lasso | 0.35 | 8.60E-36 |
| PTPN11 | 12 | rs10850031 | 0.020039 | 58 | blup | 0.025 | 0.0012 |
| ALDH2 | 12 | rs4648328 | 0.122598 | 61 | lasso | 0.13 | 9.00E-13 |
| MRVI1 | 11 | rs753002 | 0.013666 | 211 | bslmm | 0.049 | 0.0000081 |
| MADD | 11 | rs11570115 | 0.211507 | 99 | bslmm | 0.24 | 5.70E-24 |
| CSPG4 | 15 | rs4503758 | -0.00249 | 60 | blup | 0.015 | 0.01 |
| pQTL.R^2^, cross-validation R^2^ of the best pQTL in the locus; MODEL, Best performing model; MODELCV.R^2^, cross-validation R^2^ of the best performing model; MODELCV.PV, cross-validation P-value of the best performing model. | | | | | | | |

| Table S2. The PWAS of ischemic stroke integrating the ischemic stroke GWAS (N=60,341) with ROS/MAP human brain proteomic and genetic data (N=376) using FUSION. | | | | | | | | | | | | | | |
| --- | --- | --- | --- | --- | --- | --- | --- | --- | --- | --- | --- | --- | --- | --- |
| **Gene** | | **CHR** | **HSQ** | **BEST.GWAS.ID** | **BEST.GWAS.Z** | **pQTL.ID** | **pQTL.R^2^** | **pQTL.Z** | **pQTL.GWAS.Z** | **NSNP** | **NWGT** | **PWAS.Z** | **PWAS.P** | **PWAS.FDR.Q** |
| PTPN11 | 12 | | 0.0303 | rs11066301 | 6.57 | rs10850031 | 0.02 | 4.05 | -4.811 | 58 | 58 | -4.9007 | 9.55E-07 | 0.00135 |
| SLC44A2 | 19 | | 0.0264 | rs1053007 | -5.51 | rs11085744 | 0.0151 | 4.07 | -3.776 | 85 | 85 | -4.7706 | 1.84E-06 | 0.00135 |
| ALDH2 | 12 | | 0.0594 | rs2238151 | -5.79 | rs4648328 | 0.1226 | -7.25 | -4.236 | 61 | 9 | 4.3329 | 1.47E-05 | 0.00717 |
| HSD17B12 | 11 | | 0.2169 | rs10400343 | 4.5 | rs1061810 | 0.1555 | -8.14 | 4.01 | 164 | 13 | -4.0046 | 6.21E-05 | 0.02273 |
| HSQ, refers to heritability of the protein using common variants. NSNP, indicates the total number of SNPs within 200kb window (100kb up and downstream) of the gene. NWGT, refers to the total number of SNPs that contribute to the weight of the protein. | | | | | | | | | | | | | | |

| Table S3. The PWAS of large-artery atherosclerotic stroke integrating the large-artery atherosclerotic stroke GWAS (N=6,688) with ROS/MAP human brain proteomic and genetic data (N=376) using FUSION. | | | | | | | | | | | | | |
| --- | --- | --- | --- | --- | --- | --- | --- | --- | --- | --- | --- | --- | --- |
| **Gene** | **CHR** | **HSQ** | **BEST.GWAS.ID** | **BEST.GWAS.Z** | **pQTL.ID** | **pQTL.R^2^** | **pQTL.Z** | **pQTL.GWAS.Z** | **NSNP** | **NWGT** | **PWAS.Z** | **PWAS.P** | **PWAS.FDR.Q** |
| VAMP1 | 12 | 0.076 | rs2041375 | 3.62 | rs12964 | 0.045 | -3.42 | -2.504 | 107 | 107 | 3.64989 | 0.00026 | 0.2491 |
| FNBP1 | 9 | 0.099 | rs10115241 | 3.711 | rs4240438 | 0.083 | 4.22 | 3.461 | 136 | 1 | 3.461 | 0.00054 | 0.2491 |
| COX11 | 17 | 0.119 | rs17817829 | 4.04 | rs12936957 | 0.104 | -5.23 | 3.39 | 102 | 102 | -3.40785 | 0.00066 | 0.2491 |
| MAP1S | 19 | 0.26 | rs12973539 | 4.16 | rs12979056 | 0.197 | -5.82 | 2.978 | 93 | 6 | -3.2787 | 0.00104 | 0.2967 |
| HSQ, refers to heritability of the protein using common variants. NSNP, indicates the total number of SNPs within 200kb window (100kb up and downstream) of the gene. NWGT, refers to the total number of SNPs that contribute to the weight of the protein. | | | | | | | | | | | | | |

| Table S4. The PWAS of brain microbleeds integrating the brain microbleeds GWAS (N=3,556) with ROS/MAP human brain proteomic and genetic data (N=376) using FUSION. | | | | | | | | | | | | | |
| --- | --- | --- | --- | --- | --- | --- | --- | --- | --- | --- | --- | --- | --- |
| **Gene** | **CHR** | **HSQ** | **BEST.GWAS.ID** | **BEST.GWAS.Z** | **pQTL.ID** | **pQTL.R^2^** | **pQTL.Z** | **pQTL.GWAS.Z** | **NSNP** | **NWGT** | **PWAS.Z** | **PWAS.P** | **PWAS.FDR.Q** |
| RCHY1 | 4 | 0.0813 | rs11099595 | 3.43 | rs1478174 | 0.06234 | -5.16 | 3.352 | 85 | 85 | -3.7544 | 0.000174 | 0.145071 |
| C4A | 6 | 0.0918 | rs497309 | -2.96 | rs4151651 | 0.029706 | 3.64 | 2.229 | 70 | 70 | 3.7198 | 0.000199 | 0.145071 |
| MLF1 | 3 | 0.0638 | rs16847135 | 3.72 | rs7631310 | 0.024501 | -4.97 | -2.831 | 85 | 14 | 3.4348 | 0.000593 | 0.288198 |
| PDDC1 | 11 | 0.0412 | rs7937869 | 3.45 | rs12223324 | 0.014122 | 3.78 | -2.714 | 104 | 104 | -3.2504 | 0.00115 | 0.419175 |
| HSQ, refers to heritability of the protein using common variants. NSNP, indicates the total number of SNPs within 200kb window (100kb up and downstream) of the gene. NWGT, refers to the total number of SNPs that contribute to the weight of the protein. | | | | | | | | | | | | | |

| Table S5. The PWAS of neuroticism integrating the neuroticism GWAS (N=390,278) with ROS/MAP human brain proteomic and genetic data (N=376) using FUSION. | | | | | | | | | | | | | |
| --- | --- | --- | --- | --- | --- | --- | --- | --- | --- | --- | --- | --- | --- |
| **Gene** | **CHR** | **HSQ** | **BEST.GWAS.ID** | **BEST.GWAS.Z** | **pQTL.ID** | **pQTL.R^2^** | **pQTL.Z** | **pQTL.GWAS.Z** | **NSNP** | **NWGT** | **PWAS.Z** | **PWAS.P** | **PWAS.FDR.Q** |
| CTNND1 | 11 | 0.03 | rs7117205 | 6.47 | rs499188 | 0.02887 | -3.73 | 6.367 | 60 | 60 | -6.379 | 1.78E-10 | 2.61E-07 |
| AATK | 17 | 0.05 | rs4969391 | -6.99 | rs7225916 | NA | 0 | 1.629 | 85 | 85 | 5.898 | 3.69E-09 | 2.70E-06 |
| ORC4 | 2 | 0.02 | rs7582403 | 7.41 | rs4419186 | NA | 0 | 6.011 | 62 | 62 | -5.72 | 1.06E-08 | 4.29E-06 |
| CACNA2D2 | 3 | 0.09 | rs2073498 | 5.47 | rs2071803 | 0.04085 | -4.45 | 5.433 | 86 | 10 | -5.704 | 1.17E-08 | 4.29E-06 |
| STX4 | 16 | 0.04 | rs11865038 | -6.49 | rs7294 | 0.02434 | -3.78 | -6.421 | 66 | 66 | 5.658 | 1.53E-08 | 4.49E-06 |
| MMAB | 12 | 0.3 | rs11067376 | 7.52 | rs2302706 | 0.4661 | -13.65 | 5.19 | 112 | 41 | -5.61 | 1.98E-08 | 4.84E-06 |
| SLC7A8 | 14 | 0.16 | rs3783436 | -5.26 | rs3783436 | 0.13661 | -7.35 | -5.264 | 112 | 3 | 5.222 | 1.77E-07 | 3.71E-05 |
| B3GALTL | 13 | 0.24 | rs12585794 | 4.87 | rs17620350 | NA | 0 | 0.416 | 177 | 7 | -5.16 | 2.47E-07 | 4.53E-05 |
| TMEM33 | 4 | 0.04 | rs10433709 | -4.98 | rs6821891 | 0.03842 | -4.19 | -3.787 | 77 | 77 | 4.883 | 1.04E-06 | 0.00016 |
| POR | 7 | 0.37 | rs3815455 | -4.05 | rs10954732 | 0.05785 | 5.05 | -4.023 | 73 | 14 | -4.876 | 1.08E-06 | 0.00016 |
| TKT | 3 | 0.11 | rs3736151 | 4.21 | rs3736151 | 0.09241 | 6.22 | 4.212 | 93 | 21 | 4.765 | 1.89E-06 | 0.00025 |
| DCC | 18 | 0.1 | rs4632195 | 6.46 | rs1394466 | 0.072 | -6.38 | -4.391 | 648 | 648 | 4.741 | 2.13E-06 | 0.00026 |
| KCTD10 | 12 | 0.05 | rs3742020 | 8.11 | rs1477117 | 0.00394 | 3.82 | 5.305 | 120 | 120 | 4.72 | 2.33E-06 | 0.00026 |
| CSDC2 | 22 | 0.15 | rs5758365 | 5.54 | rs5758365 | NA | 0 | 5.542 | 42 | 42 | 4.695 | 2.67E-06 | 0.00028 |
| C1orf123 | 1 | 0.08 | rs3736118 | -4.664 | rs2297656 | 0.09236 | -6.46 | -4.565 | 109 | 2 | 4.585 | 4.55E-06 | 0.00044 |
| TAOK3 | 12 | 0.03 | rs7294498 | 5.96 | rs16948204 | 0.01275 | -3.95 | 5.554 | 107 | 107 | -4.54 | 5.60E-06 | 0.00051 |
| PANK4 | 1 | 0.06 | rs4648639 | -4.308 | rs10910082 | 0.0459 | 5.53 | -4.297 | 96 | 96 | -4.512 | 6.43E-06 | 0.00055 |
| RAB27B | 18 | 0.11 | rs3737477 | -4.5 | rs2871673 | 0.153 | -7.92 | -3.914 | 93 | 19 | 4.449 | 8.61E-06 | 0.0007 |
| MADD | 11 | 0.44 | rs11039149 | 7.67 | rs11570115 | 0.21151 | -9.09 | 3.809 | 99 | 99 | -4.436 | 9.16E-06 | 0.00071 |
| RGS6 | 14 | 0.12 | rs36424 | -4.94 | rs36341 | 0.06614 | -5.56 | 4.332 | 550 | 550 | -4.41 | 1.03E-05 | 0.00075 |
| PML | 15 | 0.06 | rs4558370 | -4.351 | rs9944214 | -0.00266 | -3.21 | -2.965 | 84 | 84 | 4.287 | 1.81E-05 | 0.00126 |
| DEPTOR | 8 | 0.11 | rs4871827 | -4.12 | rs7833407 | NA | 0 | 0.942 | 178 | 5 | -4.1 | 4.14E-05 | 0.00276 |
| CAPG | 2 | 0.18 | rs4247303 | -3.6 | rs3770102 | 0.11122 | 6.75 | 2.641 | 88 | 88 | 4.076 | 4.59E-05 | 0.00283 |
| NEK4 | 3 | 0.06 | rs11177 | -4.82 | rs13083798 | NA | 0 | 3.714 | 112 | 112 | 4.073 | 4.64E-05 | 0.00283 |
| CNNM2 | 10 | 0.13 | rs1004467 | -4.9 | rs619824 | NA | 0 | -3.919 | 132 | 9 | -3.957 | 7.58E-05 | 0.00444 |
| SPRYD4 | 12 | 0.17 | rs774045 | 4.48 | rs1043011 | 0.12839 | 7.13 | 3.6 | 64 | 8 | 3.95 | 7.98E-05 | 0.0045 |
| SLC30A9 | 4 | 0.08 | rs7682049 | -5.41 | rs7682049 | 0.02144 | 4.88 | -5.407 | 116 | 17 | -3.926 | 8.63E-05 | 0.00469 |
| PRKCA | 17 | 0.09 | rs9908167 | -5.39 | rs6504459 | 0.0349 | 5.28 | -2.446 | 386 | 16 | -3.842 | 0.000122 | 0.00639 |
| SGTB | 5 | 0.09 | rs42468 | 4.79 | rs1549192 | 0.08505 | -6.08 | -3.695 | 122 | 3 | 3.785 | 0.000154 | 0.00778 |
| EPHB2 | 1 | 0.08 | rs158771 | 3.505 | rs12723359 | 0.0408 | -5.01 | 2.119 | 172 | 172 | -3.766 | 0.000166 | 0.00811 |
| SLC25A12 | 2 | 0.06 | rs12692974 | 4.18 | rs6738445 | 0.05831 | 5.57 | 4.095 | 93 | 93 | 3.692 | 0.000222 | 0.0105 |
| PDIA3 | 15 | 0.04 | rs654276 | -3.664 | rs12443084 | 0.03177 | -3.81 | -3.22 | 49 | 49 | 3.654 | 0.000259 | 0.01187 |
| SHMT1 | 17 | 0.28 | rs2245737 | -4.49 | rs2461838 | 0.315 | 10.95 | 2.656 | 58 | 4 | 3.612 | 0.000303 | 0.01346 |
| LMBRD1 | 6 | 0.06 | rs9364048 | 3.54 | rs2757756 | NA | 0 | -0.791 | 130 | 11 | -3.588 | 0.000334 | 0.0144 |
| TECPR1 | 7 | 0.28 | rs11768309 | -4.78 | rs6969321 | 0.10718 | -6.66 | -3.118 | 90 | 7 | 3.561 | 0.00037 | 0.01527 |
| TMEM106B | 7 | 0.09 | rs3807866 | 6.07 | rs6945902 | 0.07164 | 5.49 | 3.094 | 139 | 12 | 3.557 | 0.000375 | 0.01527 |
| CALU | 7 | 0.08 | rs339099 | -3.69 | rs1043550 | 0.1105 | 6.63 | -3.474 | 110 | 4 | -3.537 | 0.000405 | 0.01605 |
| TGOLN2 | 2 | 0.05 | rs4247303 | -3.6 | rs4247303 | 0.09149 | -6.21 | -3.605 | 112 | 6 | 3.519 | 0.000434 | 0.01674 |
| DCAF6 | 1 | 0.13 | rs275143 | 3.721 | rs1034464 | NA | 0 | 0.67 | 108 | 3 | 3.508 | 0.000451 | 0.01695 |
| CISD1 | 10 | 0.04 | rs2790241 | 3.45 | rs714113 | 0.03336 | 4.16 | 2.276 | 88 | 88 | 3.493 | 0.000477 | 0.0172 |
| EMB | 5 | 0.09 | rs7378983 | -4.63 | rs7448495 | NA | 0 | -2.91 | 53 | 53 | -3.491 | 0.000481 | 0.0172 |
| ICA1L | 2 | 0.12 | rs10048682 | 3.93 | rs1541853 | 0.11577 | 7.21 | -3.427 | 81 | 6 | -3.479 | 0.000503 | 0.01756 |
| FLOT2 | 17 | 0.31 | rs12150102 | -4.25 | rs6505095 | 0.145 | -7.45 | -3.553 | 68 | 6 | 3.473 | 0.000515 | 0.01756 |
| FAIM2 | 12 | 0.06 | rs766977 | 3.35 | rs17123981 | NA | 0 | -0.232 | 105 | 6 | 3.46 | 0.000538 | 0.01793 |
| NUDCD1 | 8 | 0.3 | rs7012304 | -3.87 | rs7012304 | 0.07181 | 6.11 | -3.87 | 90 | 90 | -3.448 | 0.000564 | 0.01836 |
| CDK18 | 1 | 0.22 | rs734773 | -3.287 | rs12058761 | 0.01596 | -5 | -2.108 | 119 | 119 | 3.442 | 0.000576 | 0.01836 |
| BOLA1 | 1 | 0.18 | rs16835135 | -3.908 | rs15931 | 0.24748 | 10.09 | -3.431 | 24 | 3 | -3.431 | 0.000602 | 0.01878 |
| PANK2 | 20 | 0.17 | rs7262903 | 4 | rs16989000 | 0.14813 | 7.6 | 2.032 | 104 | 104 | 3.417 | 0.000634 | 0.01936 |
| SH3GL1 | 19 | 0.04 | rs243352 | 3.85 | rs760369 | 0.04246 | 4.46 | -3.23 | 118 | 9 | -3.403 | 0.000666 | 0.01993 |
| ACYP1 | 14 | 0.07 | rs12431669 | -4.17 | rs7156328 | 0.10906 | 6.55 | -3.381 | 69 | 2 | -3.379 | 0.000727 | 0.02132 |
| ADCY9 | 16 | 0.11 | rs2531982 | -3.56 | rs2230739 | 0.06753 | -5.23 | -3.339 | 179 | 4 | 3.37 | 0.000752 | 0.02162 |
| GSS | 20 | 0.04 | rs11546155 | 5.43 | rs2236271 | 0.0371 | -5.03 | -2.638 | 78 | 78 | 3.3 | 0.000967 | 0.02713 |
| P2RX7 | 12 | 0.57 | rs504677 | -3.34 | rs3751143 | 0.35375 | -11.59 | 3.165 | 144 | 31 | -3.3 | 0.000981 | 0.02713 |
| PACSIN3 | 11 | 0.07 | rs11039149 | 7.67 | rs7946709 | NA | 0 | -5.279 | 83 | 83 | -3.263 | 0.0011 | 0.02932 |
| DNAJC10 | 2 | 0.05 | rs288316 | -4.24 | rs415994 | 0.00951 | -3.55 | -2.11 | 113 | 113 | 3.264 | 0.0011 | 0.02932 |
| FAM160B2 | 8 | 0.03 | rs2309308 | -4.13 | rs17296501 | NA | 0 | 1.293 | 91 | 3 | -3.245 | 0.00118 | 0.03089 |
| TMEM160 | 19 | 0.09 | rs8101491 | 3.77 | rs311384 | NA | 0 | 1.947 | 36 | 7 | -3.229 | 0.001244 | 0.03159 |
| TMEM25 | 11 | 0.08 | rs9332809 | 3.9 | rs1939950 | NA | 0 | 0.006 | 67 | 67 | -3.226 | 0.00125 | 0.03159 |
| GPX1 | 3 | 0.29 | rs4955417 | 3.88 | rs17080528 | 0.33445 | -11.25 | -3.032 | 52 | 10 | 3.218 | 0.00129 | 0.03205 |
| AMPH | 7 | 0.09 | rs11765454 | -3.95 | rs1019288 | 0.04316 | 4.38 | -1.693 | 304 | 304 | -3.211 | 0.00132 | 0.03225 |
| CNTN5 | 11 | 0.21 | rs658789 | 4.91 | rs17660476 | 0.08277 | -5.79 | 3.397 | 870 | 870 | -3.175 | 0.0015 | 0.03605 |
| LYRM9 | 17 | 0.15 | rs8067428 | 3.58 | rs7218395 | 0.262 | -10.27 | 3.054 | 75 | 3 | -3.163 | 0.00156 | 0.03689 |
| UBE3B | 12 | 0.07 | rs3742020 | 8.11 | rs7358739 | 0.04064 | 4.44 | 3.732 | 131 | 3 | 3.14 | 0.00168 | 0.03909 |
| FARP1 | 13 | 0.12 | rs9517301 | 4.24 | rs8002818 | NA | 0 | 0.283 | 353 | 353 | 3.125 | 0.00178 | 0.04037 |
| AAGAB | 15 | 0.03 | rs16950804 | 3.262 | rs17213990 | NA | 0 | -1.495 | 120 | 120 | -3.123 | 0.00179 | 0.04037 |
| NDUFAF2 | 5 | 0.15 | rs13183007 | 2.47 | rs11951606 | 0.07071 | 5.47 | 2.18 | 107 | 12 | 3.117 | 0.001828 | 0.0406 |
| ATG7 | 3 | 0.17 | rs347587 | -3.53 | rs346076 | 0.11958 | -7.07 | -3.295 | 200 | 200 | 3.108 | 0.00189 | 0.04135 |
| RBBP9 | 20 | 0.17 | rs2295556 | -4.93 | rs6045490 | 0.06264 | -5.97 | -3.954 | 94 | 94 | 3.079 | 0.002076 | 0.04476 |
| FASTKD5 | 20 | 0.19 | rs306755 | -4.4 | rs1034063 | NA | 0 | -1.757 | 84 | 84 | -3.071 | 0.002134 | 0.0452 |
| CDH13 | 16 | 0.25 | rs7200240 | 3.91 | rs9933518 | 0.05493 | 5.58 | -3.814 | 1385 | 1385 | -3.059 | 0.00222 | 0.0452 |
| DNM3 | 1 | 0.1 | rs7513809 | -5.169 | rs7528296 | 0.04173 | -5.38 | -1.584 | 389 | 389 | 3.059 | 0.00222 | 0.0452 |
| EFR3A | 8 | 0.04 | rs9297833 | 3.49 | rs1501061 | 0.0237 | -4.24 | 2.801 | 168 | 9 | -3.059 | 0.00222 | 0.0452 |
| HSQ, refers to heritability of the protein using common variants. NSNP, indicates the total number of SNPs within 200kb window (100kb up and downstream) of the gene. NWGT, refers to the total number of SNPs that contribute to the weight of the protein. | | | | | | | | | | | | | |

| Table S6. The PWAS of BMI integrating the BMI GWAS (N=681,275) with ROS/MAP human brain proteomic and genetic data (N=376) using FUSION. | | | | | | | | | | | | | |
| --- | --- | --- | --- | --- | --- | --- | --- | --- | --- | --- | --- | --- | --- |
| **Gene** | **CHR** | **HSQ** | **BEST.GWAS.ID** | **BEST.GWAS.Z** | **pQTL.ID** | **pQTL.R^2^** | **pQTL.Z** | **pQTL.GWAS.Z** | **NSNP** | **NWGT** | **PWAS.Z** | **PWAS.P** | **PWAS.FDR.Q** |
| ADCY3 | 2 | 0.0378 | rs10182181 | 20.31 | rs6719275 | NA | 0 | 0 | 121 | 121 | -15.4875 | 4.21E-54 | 6.07E-51 |
| DOC2A | 16 | 0.0447 | rs11649274 | 13.01 | rs11642612 | 0.03385 | 4.56 | 12.4444 | 74 | 74 | 13.5996 | 4.03E-42 | 2.91E-39 |
| SULT1A2 | 16 | 0.3316 | rs4788084 | 15.69 | rs27741 | NA | 0 | 14 | 51 | 11 | -12.3606 | 4.27E-35 | 2.05E-32 |
| SULT1A1 | 16 | 0.2801 | rs4788084 | 15.69 | rs1968752 | 0.10631 | -7.07 | 14.7059 | 45 | 10 | -11.6522 | 2.24E-31 | 8.08E-29 |
| CNNM2 | 10 | 0.1255 | rs12411886 | 9.03 | rs619824 | NA | 0 | 6.6875 | 132 | 9 | 10.6629 | 1.52E-26 | 4.38E-24 |
| LMOD1 | 1 | 0.0479 | rs2820313 | 13.17 | rs7551784 | NA | 0 | -7.4444 | 105 | 105 | -10.6367 | 2.01E-26 | 4.83E-24 |
| HSD17B12 | 11 | 0.2169 | rs7928523 | 11.83 | rs1061810 | 0.15547 | -8.14 | 11 | 164 | 13 | -9.2269 | 2.79E-20 | 5.75E-18 |
| DHRS11 | 17 | 0.1559 | rs1106908 | -9.88 | rs35712149 | 0.28 | -10.45 | -9.4606 | 101 | 18 | 9.1469 | 5.86E-20 | 1.06E-17 |
| RAB27B | 18 | 0.114 | rs8092503 | 8.68 | rs2871673 | 0.153 | -7.92 | 8.45 | 93 | 19 | -8.574 | 1.00E-17 | 1.60E-15 |
| MADD | 11 | 0.4422 | rs755553 | 13.53 | rs11570115 | 0.21151 | -9.09 | -6.6296 | 99 | 99 | 8.4039 | 4.32E-17 | 6.23E-15 |
| SUGP1 | 19 | 0.065 | rs998732 | -7.77 | rs539 | NA | 0 | 0.5294 | 83 | 12 | -8.16 | 3.36E-16 | 4.40E-14 |
| SNX32 | 11 | 0.1683 | rs2303385 | 9.01 | rs2231884 | 0.18336 | 8.7 | 7.3323 | 86 | 5 | 7.6465 | 2.07E-14 | 2.49E-12 |
| TRAF3 | 14 | 0.1043 | rs3803286 | 10.06 | rs3803286 | 0.05062 | 4.95 | 10.0556 | 102 | 102 | 7.6168 | 2.60E-14 | 2.88E-12 |
| GPX1 | 3 | 0.2944 | rs10640 | -7.63 | rs17080528 | 0.33445 | -11.25 | -7.5659 | 52 | 10 | 7.50538 | 6.13E-14 | 6.31E-12 |
| ASPHD1 | 16 | 0.168 | rs7190185 | 12.95 | rs34286592 | NA | 0 | 7.5581 | 67 | 67 | 7.4523 | 9.17E-14 | 8.82E-12 |
| RASA2 | 3 | 0.1021 | rs16851483 | 10.54 | rs16851483 | 0.08467 | 6.12 | 10.5429 | 138 | 138 | 7.28579 | 3.20E-13 | 2.88E-11 |
| HIP1R | 12 | 0.134 | rs11060180 | -7.06 | rs11060180 | 0.05238 | 4.6 | -7.0556 | 65 | 3 | -7.205 | 5.81E-13 | 4.93E-11 |
| SLC25A12 | 2 | 0.0587 | rs6738445 | -7.39 | rs6738445 | 0.05831 | 5.57 | -7.3889 | 93 | 93 | -7.1698 | 7.51E-13 | 6.02E-11 |
| ADCY9 | 16 | 0.1143 | rs879620 | -12.83 | rs2230739 | 0.06753 | -5.23 | 6.0526 | 179 | 4 | -7.1356 | 9.64E-13 | 7.32E-11 |
| PLCL1 | 2 | 0.0682 | rs16826873 | -7.59 | rs1595823 | 0.06886 | 5.79 | -6.3529 | 180 | 10 | -7.0524 | 1.76E-12 | 1.27E-10 |
| RASGRF1 | 15 | 0.0436 | rs12595749 | -8.29 | rs12595749 | 0.03777 | -4.2 | -8.2941 | 164 | 164 | 6.8964 | 5.33E-12 | 3.66E-10 |
| NRBF2 | 10 | 0.0363 | rs4379723 | -6.65 | rs10995404 | NA | 0 | -5.0714 | 77 | 2 | 6.8747 | 6.21E-12 | 4.07E-10 |
| ABCG2 | 4 | 0.1007 | rs4148155 | -7.23 | rs12650232 | NA | 0 | -0.538 | 127 | 2 | 6.8148 | 9.44E-12 | 5.92E-10 |
| PLCB3 | 11 | 0.033 | rs34882006 | -8.41 | rs11603192 | 0.00062 | 3.43 | -3.7222 | 81 | 6 | -6.7852 | 1.16E-11 | 6.97E-10 |
| DPYSL4 | 10 | 0.0641 | rs7096307 | 6.94 | rs7096307 | 0.07075 | 5.42 | 6.9412 | 125 | 2 | 6.7285 | 1.71E-11 | 9.86E-10 |
| FAM114A2 | 5 | 0.0609 | rs12054772 | 8.88 | rs378267 | 0.10451 | -6.44 | -6.5556 | 90 | 14 | 6.70313 | 2.04E-11 | 1.10E-09 |
| ADPGK | 15 | 0.1077 | rs7164727 | -10.71 | rs12441929 | NA | 0 | -1.7214 | 67 | 4 | 6.7019 | 2.06E-11 | 1.10E-09 |
| CDC42BPB | 14 | 0.0778 | rs7143963 | 8.95 | rs1190234 | 0.02119 | 4.51 | 6.5385 | 99 | 4 | 6.6297 | 3.36E-11 | 1.73E-09 |
| SNX19 | 11 | 0.0727 | rs10894294 | -8.19 | rs6590507 | NA | 0 | -1.0312 | 145 | 145 | 6.5362 | 6.31E-11 | 3.14E-09 |
| ITGAM | 16 | 0.0213 | rs1052352 | 8.18 | rs4075052 | 0.01957 | -3.65 | 5.5 | 76 | 76 | -6.4716 | 9.70E-11 | 4.66E-09 |
| DGKG | 3 | 0.0775 | rs6809651 | -15.04 | rs1004589 | 0.00769 | 3.73 | 1.1667 | 201 | 201 | -6.45313 | 1.10E-10 | 5.07E-09 |
| CRTAC1 | 10 | 0.0991 | rs522110 | -9.71 | rs7922066 | 0.08597 | -5.83 | 5.0454 | 169 | 13 | -6.448 | 1.13E-10 | 5.07E-09 |
| CCDC92 | 12 | 0.0507 | rs7133378 | 6.94 | rs12309481 | 0.0581 | -5.61 | 3.2778 | 83 | 83 | -6.445 | 1.16E-10 | 5.07E-09 |
| ULK3 | 15 | 0.1771 | rs936227 | -6.94 | rs936227 | 0.25597 | 9.94 | -6.9412 | 87 | 8 | -6.4205 | 1.36E-10 | 5.77E-09 |
| C3orf18 | 3 | 0.0602 | rs1034405 | -6.4 | rs2236984 | NA | 0 | -3.52 | 49 | 8 | -6.40822 | 1.47E-10 | 6.06E-09 |
| NASP | 1 | 0.0201 | rs4660880 | -6.72 | rs3014210 | 0.02681 | -3.79 | -5.4 | 84 | 84 | 6.35045 | 2.15E-10 | 8.61E-09 |
| SPRYD4 | 12 | 0.1706 | rs774211 | -6.32 | rs1043011 | 0.12839 | 7.13 | -5.9091 | 64 | 8 | -6.329 | 2.47E-10 | 9.63E-09 |
| CSE1L | 20 | 0.0632 | rs10485609 | -6.9 | rs1556876 | 0.05422 | -5.02 | -6.9 | 79 | 6 | 6.2975 | 3.03E-10 | 1.15E-08 |
| TRMT61B | 2 | 0.19 | rs4372836 | 7.89 | rs4372836 | NA | 0 | 7.8889 | 74 | 3 | 6.1928 | 5.91E-10 | 2.19E-08 |
| TTC19 | 17 | 0.0322 | rs1075901 | -7.56 | rs758853 | 0.0281 | -3.88 | -6.2941 | 73 | 5 | 5.9642 | 2.46E-09 | 8.76E-08 |
| DNM1 | 9 | 0.0381 | rs12005136 | 7.52 | rs2302425 | 0.01028 | 3.58 | 1.7105 | 81 | 81 | 5.9621 | 2.49E-09 | 8.76E-08 |
| TRIM47 | 17 | 0.0673 | rs9894383 | 5.73 | rs1060120 | NA | 0 | -4.2665 | 62 | 6 | -5.8946 | 3.76E-09 | 1.29E-07 |
| ST5 | 11 | 0.1195 | rs10840100 | -10.71 | rs4450173 | NA | 0 | -2.3636 | 124 | 11 | -5.8823 | 4.05E-09 | 1.36E-07 |
| NEK4 | 3 | 0.0616 | rs2710323 | -8.81 | rs13083798 | NA | 0 | -8.4194 | 112 | 112 | -5.77745 | 7.58E-09 | 2.48E-07 |
| MACF1 | 1 | 0.0414 | rs2282231 | 7.86 | rs1539435 | 0.0312 | -4.18 | -5.7586 | 203 | 1 | 5.75862 | 8.48E-09 | 2.66E-07 |
| PPTC7 | 12 | 0.0545 | rs4766500 | 7.41 | rs1502337 | 0.02773 | -4.61 | 7.4118 | 50 | 11 | -5.758 | 8.50E-09 | 2.66E-07 |
| TUBA4A | 2 | 0.0247 | rs7600417 | -7.83 | rs4485556 | 0.00018 | -3.01 | -7.3182 | 87 | 87 | 5.7158 | 1.09E-08 | 3.34E-07 |
| ARL3 | 10 | 0.1608 | rs4146429 | 8.03 | rs2298278 | 0.01235 | -4.39 | 7.8276 | 100 | 100 | -5.6966 | 1.22E-08 | 3.67E-07 |
| COMT | 22 | 0.3762 | rs4680 | -5.625 | rs11089314 | NA | 0 | -0.087 | 178 | 3 | -5.691 | 1.26E-08 | 3.71E-07 |
| SRR | 17 | 0.1658 | rs7217226 | 7.65 | rs12450028 | 0.192 | -8.8 | -5.6847 | 112 | 1 | 5.6847 | 1.31E-08 | 3.78E-07 |
| NAT6 | 3 | 0.0792 | rs2236941 | 9.29 | rs34877766 | NA | 0 | 6.513 | 52 | 3 | 5.6302 | 1.80E-08 | 5.09E-07 |
| EPHB2 | 1 | 0.0819 | rs6692586 | 8.35 | rs12723359 | 0.0408 | -5.01 | -4.4444 | 172 | 172 | 5.58561 | 2.33E-08 | 6.46E-07 |
| DNAJC11 | 1 | 0.1764 | rs2235564 | 7.28 | rs200448 | 0.04855 | 5.23 | 6.4706 | 109 | 13 | 5.53155 | 3.17E-08 | 8.62E-07 |
| SLC9A3R2 | 16 | 0.0959 | rs2516739 | -7.57 | rs7185040 | 0.03115 | -4.47 | -5.2273 | 63 | 21 | 5.498 | 3.84E-08 | 1.03E-06 |
| HINT1 | 5 | 0.0801 | rs1035440 | -5.71 | rs3891636 | 0.07713 | 5.5 | -5.5 | 67 | 3 | -5.49423 | 3.92E-08 | 1.03E-06 |
| CISD2 | 4 | 0.0707 | rs223391 | -6.24 | rs223452 | 0.09964 | -6.51 | -4.647 | 43 | 3 | 5.489 | 4.04E-08 | 1.04E-06 |
| CMPK1 | 1 | 0.4749 | rs6700838 | 10 | rs7555040 | 0.11771 | -6.83 | 2.9167 | 85 | 14 | -5.48634 | 4.10E-08 | 1.04E-06 |
| C16orf62 | 16 | 0.0672 | rs2354584 | -11.15 | rs11642806 | 0.00305 | -3.29 | 3.5 | 143 | 143 | -5.45 | 5.04E-08 | 1.25E-06 |
| TYW5 | 2 | 0.0451 | rs166849 | -5.41 | rs769957 | NA | 0 | -4.3684 | 75 | 3 | 5.4389 | 5.36E-08 | 1.31E-06 |
| PSMD9 | 12 | 0.1108 | rs1169084 | -5.74 | rs1169081 | 0.08971 | -6.52 | -4.8947 | 89 | 10 | 5.434 | 5.50E-08 | 1.31E-06 |
| RABEP1 | 17 | 0.0807 | rs1000940 | 8.56 | rs9891728 | 0.0961 | -6.64 | -5.0556 | 117 | 3 | 5.4326 | 5.55E-08 | 1.31E-06 |
| VPS13C | 15 | 0.1803 | rs12595158 | -7.3 | rs12442569 | 0.05218 | -6.74 | -6.9 | 205 | 4 | 5.4243 | 5.82E-08 | 1.35E-06 |
| FUT8 | 14 | 0.0315 | rs17753508 | 5.14 | rs10132229 | NA | 0 | -1.6207 | 172 | 172 | -5.4098 | 6.31E-08 | 1.44E-06 |
| ACP1 | 2 | 0.2398 | rs6724817 | 7.59 | rs9213 | 0.35606 | -11.7 | -5.3889 | 96 | 6 | 5.3893 | 7.07E-08 | 1.59E-06 |
| APMAP | 20 | 0.0392 | rs6138482 | 7.35 | rs6138478 | 0.02585 | -4.43 | -5.0118 | 120 | 2 | 5.3828 | 7.33E-08 | 1.63E-06 |
| GOLGA3 | 12 | 0.098 | rs7136785 | 7.05 | rs5744773 | NA | 0 | -1.9259 | 101 | 101 | -5.271 | 1.36E-07 | 2.97E-06 |
| CPNE3 | 8 | 0.0872 | rs7006629 | -6.41 | rs10956871 | 0.07167 | -5.38 | 4.5263 | 102 | 102 | -5.2352 | 1.65E-07 | 3.54E-06 |
| RAB23 | 6 | 0.0486 | rs11398 | 4.74 | rs9382684 | 0.02351 | -4.11 | -3.5674 | 93 | 27 | 5.233 | 1.67E-07 | 3.54E-06 |
| OLA1 | 2 | 0.0485 | rs17239377 | 8.06 | rs9332424 | 0.01812 | 4.29 | 6 | 159 | 16 | 5.2181 | 1.81E-07 | 3.78E-06 |
| ERO1L | 14 | 0.0284 | rs10144318 | -5.75 | rs12892461 | 0.02435 | 3.78 | -5.1091 | 77 | 77 | -5.2095 | 1.89E-07 | 3.89E-06 |
| ERAP1 | 5 | 0.3732 | rs3822683 | -6.9 | rs41135 | 0.25961 | -10.8 | 3.1176 | 174 | 28 | -5.19996 | 1.99E-07 | 4.04E-06 |
| UBQLN4 | 1 | 0.0534 | rs11577179 | -6.35 | rs1111102 | 0.02496 | -4.01 | -4.2632 | 73 | 3 | 5.14051 | 2.74E-07 | 5.49E-06 |
| PCYT1A | 3 | 0.0533 | rs6764533 | 6.44 | rs884534 | 0.04253 | -4.94 | -3.9 | 101 | 4 | 5.09812 | 3.43E-07 | 6.74E-06 |
| ACOT8 | 20 | 0.1988 | rs7270170 | 4.12 | rs7270170 | 0.09643 | 6.32 | 4.125 | 103 | 20 | 5.0965 | 3.46E-07 | 6.74E-06 |
| LUZP1 | 1 | 0.0582 | rs4655141 | 8.35 | rs683893 | 0.01678 | 3.94 | 8.0985 | 97 | 97 | 5.0881 | 3.62E-07 | 6.96E-06 |
| SENP8 | 15 | 0.0857 | rs12442886 | -5.6 | rs2957740 | NA | 0 | -4.6842 | 57 | 12 | 5.0676 | 4.03E-07 | 7.65E-06 |
| AK5 | 1 | 0.0864 | rs6695572 | 11 | rs728741 | 0.0862 | 5.97 | -2.8095 | 265 | 265 | -5.054 | 4.33E-07 | 8.11E-06 |
| AATK | 17 | 0.0452 | rs4075483 | -7.5 | rs7225916 | NA | 0 | 1.8 | 85 | 85 | -5.0413 | 4.62E-07 | 8.54E-06 |
| PDXDC1 | 16 | 0.0414 | rs4985155 | -7.06 | rs4985155 | 0.00328 | -3.69 | -7.0588 | 31 | 31 | 5.0193 | 5.19E-07 | 9.47E-06 |
| H1F0 | 22 | 0.0247 | rs2285178 | 5.895 | rs4821714 | 0.00888 | 3.3 | 4.9474 | 58 | 58 | 5.016 | 5.27E-07 | 9.50E-06 |
| CHORDC1 | 11 | 0.0537 | rs647248 | -6.79 | rs12417707 | 0.00819 | -4 | 4.1429 | 85 | 85 | -4.9617 | 6.99E-07 | 1.24E-05 |
| ADK | 10 | 0.054 | rs7924176 | -6.82 | rs1908337 | 0.05313 | 4.95 | 4.6111 | 160 | 3 | 4.9436 | 7.67E-07 | 1.35E-05 |
| BCL2L13 | 22 | 0.1088 | rs725769 | 5.947 | rs2535708 | 0.07351 | -6.34 | 5.4286 | 149 | 6 | -4.87 | 1.12E-06 | 1.95E-05 |
| PDE2A | 11 | 0.0535 | rs7123876 | 6.26 | rs7123876 | 0.00267 | -4.01 | 6.2632 | 149 | 149 | -4.8507 | 1.23E-06 | 2.11E-05 |
| IFT27 | 22 | 0.0906 | rs2284006 | 4.9 | rs2284001 | NA | 0 | 4.6316 | 131 | 7 | -4.846 | 1.26E-06 | 2.14E-05 |
| SH3GL1 | 19 | 0.0446 | rs2705 | -5.97 | rs760369 | 0.04246 | 4.46 | 3.892 | 118 | 9 | 4.843 | 1.28E-06 | 2.15E-05 |
| WBP2 | 17 | 0.0364 | rs7209235 | 5.84 | rs7370 | 0.0235 | -4.77 | 3.9 | 59 | 6 | -4.8331 | 1.34E-06 | 2.22E-05 |
| PNPO | 17 | 0.6194 | rs2240119 | 8.41 | rs1005655 | NA | 0 | 3.9444 | 104 | 4 | 4.8307 | 1.36E-06 | 2.23E-05 |
| MRAS | 3 | 0.0388 | rs1199333 | 6.77 | rs4678405 | NA | 0 | -2.1765 | 81 | 81 | -4.80167 | 1.57E-06 | 2.53E-05 |
| EML2 | 19 | 0.0732 | rs11672660 | -16.19 | rs7252175 | 0.09066 | -6.08 | 4.28 | 86 | 4 | -4.801 | 1.58E-06 | 2.53E-05 |
| MPI | 15 | 0.1063 | rs936227 | -6.94 | rs8031937 | 0.15727 | -8.4 | -3.4444 | 79 | 10 | 4.7793 | 1.76E-06 | 2.79E-05 |
| DHX38 | 16 | 0.0476 | rs4788454 | -7.59 | rs17664900 | NA | 0 | -0.48 | 115 | 115 | 4.7657 | 1.88E-06 | 2.95E-05 |
| SERGEF | 11 | 0.1909 | rs1003921 | -4.08 | rs12420653 | NA | 0 | -0.3 | 178 | 5 | 4.7622 | 1.91E-06 | 2.96E-05 |
| DLG1 | 3 | 0.1955 | rs9876485 | 5 | rs9880331 | 0.03454 | 5.13 | -3.5926 | 209 | 209 | -4.7555 | 1.98E-06 | 3.02E-05 |
| NQO1 | 16 | 0.3444 | rs244415 | -12.18 | rs1437135 | 0.34651 | -11.99 | 4.5909 | 74 | 19 | -4.7542 | 1.99E-06 | 3.02E-05 |
| ECHDC1 | 6 | 0.0569 | rs2875982 | -5.24 | rs3823043 | 0.00164 | 3.26 | -2.0294 | 65 | 65 | -4.752 | 2.01E-06 | 3.02E-05 |
| STAT6 | 12 | 0.1076 | rs6581124 | 5.34 | rs4759275 | 0.04722 | 5.16 | 4.6667 | 78 | 78 | 4.741 | 2.12E-06 | 3.15E-05 |
| MICAL1 | 6 | 0.1675 | rs12204742 | -4.49 | rs2275650 | NA | 0 | -3.8333 | 77 | 17 | 4.71 | 2.48E-06 | 3.65E-05 |
| ATP13A2 | 1 | 0.0567 | rs761423 | -6.65 | rs2076606 | NA | 0 | 3.742 | 113 | 113 | -4.64882 | 3.34E-06 | 4.86E-05 |
| AAK1 | 2 | 0.0416 | rs6747389 | -6.89 | rs2312207 | 0.02092 | 3.98 | -6.2941 | 115 | 8 | -4.6004 | 4.22E-06 | 6.09E-05 |
| SV2C | 5 | 0.0507 | rs4490566 | -4.62 | rs12055083 | NA | 0 | 0.931 | 229 | 7 | -4.59454 | 4.34E-06 | 6.20E-05 |
| USP47 | 11 | 0.0478 | rs2054117 | 6.18 | rs10831676 | 0.05414 | -4.76 | -4.5882 | 196 | 1 | 4.5882 | 4.47E-06 | 6.32E-05 |
| KCTD5 | 16 | 0.0676 | rs12918081 | -4.58 | rs1005273 | NA | 0 | 0.9444 | 50 | 6 | -4.5841 | 4.56E-06 | 6.38E-05 |
| CERS1 | 19 | 0.044 | rs4808866 | 4.55 | rs10038 | NA | 0 | 3.1579 | 60 | 11 | -4.58 | 4.65E-06 | 6.39E-05 |
| TYK2 | 19 | 0.2406 | rs2278442 | 4.33 | rs11668618 | NA | 0 | -0.4843 | 80 | 6 | -4.58 | 4.65E-06 | 6.39E-05 |
| ZMPSTE24 | 1 | 0.0857 | rs6600323 | -4.71 | rs6682701 | 0.09358 | -6.01 | -4.5556 | 89 | 4 | 4.55488 | 5.24E-06 | 7.13E-05 |
| PIP4K2A | 10 | 0.2101 | rs1053454 | 4.89 | rs10828316 | 0.22108 | -9.21 | 4.7222 | 144 | 3 | -4.5495 | 5.38E-06 | 7.25E-05 |
| DPYSL5 | 2 | 0.1548 | rs714447 | 6.4 | rs41448746 | 0.07968 | -6.38 | -5.4886 | 87 | 87 | 4.5448 | 5.50E-06 | 7.34E-05 |
| EXOG | 3 | 0.0403 | rs7427447 | -5.84 | rs2186457 | 0.00338 | 3.24 | -5.6111 | 126 | 126 | -4.53912 | 5.65E-06 | 7.45E-05 |
| TMEM163 | 2 | 0.0329 | rs10928512 | 4.29 | rs6430538 | 0.02104 | -3.93 | -4.2778 | 204 | 204 | 4.5378 | 5.68E-06 | 7.45E-05 |
| ATG7 | 3 | 0.1745 | rs17776719 | 6.68 | rs346076 | 0.11958 | -7.07 | 4.5 | 200 | 200 | -4.51808 | 6.24E-06 | 8.11E-05 |
| ATP13A1 | 19 | 0.0932 | rs1036215 | -7.09 | rs2304130 | 0.08058 | 5.93 | -5.8667 | 94 | 3 | -4.497 | 6.88E-06 | 8.84E-05 |
| CEP41 | 7 | 0.0697 | rs12672192 | 4.89 | rs1990790 | 0.03446 | 5.04 | -4.4812 | 129 | 7 | -4.4945 | 6.97E-06 | 8.84E-05 |
| CSAD | 12 | 0.0376 | rs12368757 | 4.72 | rs2280446 | NA | 0 | 2.1917 | 85 | 85 | -4.494 | 6.99E-06 | 8.84E-05 |
| ZC3HAV1 | 7 | 0.0672 | rs11525873 | -8.57 | rs6975036 | 0.03491 | 4.35 | -5.4709 | 116 | 116 | -4.4713 | 7.77E-06 | 9.74E-05 |
| LY6H | 8 | 0.0709 | rs4570175 | 4.76 | rs10109061 | 0.04843 | -5.4 | -3.6667 | 78 | 78 | 4.4591 | 8.23E-06 | 0.000102 |
| NRP1 | 10 | 0.1089 | rs10827252 | -4.94 | rs2755979 | NA | 0 | 2.3889 | 225 | 225 | 4.4344 | 9.23E-06 | 0.000114 |
| ATG9A | 2 | 0.0295 | rs7600417 | -7.83 | rs3731900 | 0.00622 | 3.7 | -0.2941 | 88 | 88 | -4.4165 | 1.00E-05 | 0.000122 |
| SAT2 | 17 | 0.3143 | rs34416693 | -5.52 | rs9913778 | 0.219 | -9.32 | -3.1734 | 102 | 102 | 4.4158 | 1.01E-05 | 0.000122 |
| NFU1 | 2 | 0.0488 | rs10164798 | -7 | rs6724567 | 0.01305 | 3.92 | 2.6471 | 92 | 92 | 4.4124 | 1.02E-05 | 0.000123 |
| PRICKLE1 | 12 | 0.0747 | rs1796346 | -4.72 | rs11181461 | NA | 0 | 2.55 | 95 | 95 | -4.405 | 1.06E-05 | 0.000125 |
| FARS2 | 6 | 0.0943 | rs9328321 | 4.48 | rs9405807 | NA | 0 | -3.25 | 411 | 10 | 4.402 | 1.07E-05 | 0.000125 |
| GBA2 | 9 | 0.1846 | rs7873822 | 4.86 | rs3750434 | 0.20854 | -8.94 | -4.4118 | 104 | 2 | 4.4024 | 1.07E-05 | 0.000125 |
| SLC30A9 | 4 | 0.0812 | rs3810840 | 4.95 | rs7682049 | 0.02144 | 4.88 | 2.05 | 116 | 17 | 4.377 | 1.20E-05 | 0.00014 |
| TESC | 12 | 0.0605 | rs4492907 | 5.44 | rs2270794 | 0.04661 | -5.74 | 2.9243 | 103 | 103 | -4.358 | 1.31E-05 | 0.000151 |
| PTGR2 | 14 | 0.3321 | rs11621523 | -4.41 | rs8020267 | 0.21266 | 10.16 | -3.7742 | 104 | 23 | -4.3562 | 1.32E-05 | 0.000151 |
| KCTD21 | 11 | 0.0933 | rs1385601 | -5.68 | rs588584 | NA | 0 | 0.45 | 99 | 99 | -4.3085 | 1.64E-05 | 0.000186 |
| RPA1 | 17 | 0.0423 | rs4239060 | -10.45 | rs7216701 | 0.0162 | 4.31 | 2.4737 | 137 | 137 | 4.2857 | 1.82E-05 | 0.000205 |
| ACTR1B | 2 | 0.3025 | rs11683207 | 5.82 | rs11692435 | 0.13293 | -7.13 | 5.1515 | 42 | 42 | -4.2838 | 1.84E-05 | 0.000206 |
| COQ6 | 14 | 0.0781 | rs11628074 | -4.35 | rs11159052 | 0.08347 | 6.09 | 4 | 96 | 5 | 4.2792 | 1.88E-05 | 0.000209 |
| ICA1L | 2 | 0.1162 | rs3845802 | 7.19 | rs1541853 | 0.11577 | 7.21 | -4.3333 | 81 | 6 | -4.2749 | 1.91E-05 | 0.00021 |
| RASA1 | 5 | 0.2211 | rs323781 | 7.04 | rs13157168 | 0.10151 | -6.47 | -4.2286 | 69 | 69 | 4.26952 | 1.96E-05 | 0.000214 |
| KIF21B | 1 | 0.2063 | rs296565 | 3.82 | rs502658 | 0.16628 | 8.31 | 3.7273 | 151 | 14 | 4.26568 | 1.99E-05 | 0.000216 |
| RWDD1 | 6 | 0.0383 | rs6904837 | 4.21 | rs11961321 | 0.04085 | -5.1 | 4.0952 | 74 | 4 | -4.258 | 2.07E-05 | 0.000223 |
| CCDC127 | 5 | 0.3272 | rs6888378 | 4.46 | rs9763161 | 0.15093 | 7.73 | -2.7778 | 110 | 11 | -4.25055 | 2.13E-05 | 0.000228 |
| IGFBP2 | 2 | 0.0454 | rs4550645 | 3.82 | rs12612079 | NA | 0 | 0.9722 | 119 | 119 | 4.2443 | 2.19E-05 | 0.000232 |
| GNAQ | 9 | 0.0215 | rs2378100 | -5.41 | rs7856137 | 0.01913 | -4.13 | -4.2105 | 165 | 1 | 4.2105 | 2.55E-05 | 0.000268 |
| MIPEP | 13 | 0.1788 | rs10507333 | -4.4 | rs11551114 | 0.20111 | -9.61 | -4 | 232 | 7 | 4.2099 | 2.56E-05 | 0.000268 |
| ACE | 17 | 0.0783 | rs7210095 | -7.34 | rs4305 | 0.0626 | 5.51 | 3.7222 | 68 | 3 | 4.1946 | 2.73E-05 | 0.000283 |
| CACNB3 | 12 | 0.0465 | rs11168732 | 5.68 | rs12369114 | 0.03831 | -4.11 | 4.0783 | 79 | 3 | -4.192 | 2.76E-05 | 0.000284 |
| STX4 | 16 | 0.0427 | rs1549293 | -12 | rs7294 | 0.02434 | -3.78 | 6.7222 | 66 | 66 | -4.1905 | 2.78E-05 | 0.000284 |
| LYRM4 | 6 | 0.2597 | rs2224391 | -4.3 | rs9504370 | 0.30742 | -10.9 | -4 | 218 | 3 | 4.159 | 3.20E-05 | 0.000325 |
| NDUFAF1 | 15 | 0.1664 | rs316611 | 5.2 | rs17730589 | 0.17309 | -8.41 | 4.3 | 66 | 15 | -4.1546 | 3.26E-05 | 0.000329 |
| ADAP1 | 7 | 0.0529 | rs13171 | -4.79 | rs4721949 | 0.00613 | -3.61 | -4.5318 | 81 | 81 | 4.1516 | 3.30E-05 | 0.00033 |
| LRRC8B | 1 | 0.0286 | rs12745246 | -4.9 | rs9427984 | 0.00867 | -3.53 | 2.3182 | 108 | 108 | -4.14881 | 3.34E-05 | 0.000332 |
| CANX | 5 | 0.025 | rs3797774 | 4.71 | rs12652934 | 0.02382 | 3.75 | 3.9672 | 78 | 78 | 4.14601 | 3.38E-05 | 0.000334 |
| IGLON5 | 19 | 0.1273 | rs2062655 | 6.39 | rs2062655 | 0.03323 | 4.9 | 6.3889 | 113 | 113 | 4.138 | 3.50E-05 | 0.000343 |
| MKRN1 | 7 | 0.19 | rs12155423 | -4.37 | rs12155423 | 0.0818 | -6.01 | -4.3684 | 96 | 5 | 4.0746 | 4.61E-05 | 0.000447 |
| TMEM120A | 7 | 0.067 | rs6978677 | 5.74 | rs7223 | NA | 0 | 0.9424 | 73 | 8 | -4.0734 | 4.63E-05 | 0.000447 |
| TNFAIP2 | 14 | 0.134 | rs10134022 | 3.7 | rs10134022 | NA | 0 | 3.7 | 71 | 71 | -4.0727 | 4.65E-05 | 0.000447 |
| TMA16 | 4 | 0.2983 | rs2044052 | -4.76 | rs2165549 | NA | 0 | 0.5 | 115 | 5 | -4.0509 | 5.10E-05 | 0.000483 |
| SLC6A17 | 1 | 0.1327 | rs4838937 | -4.47 | rs7554444 | 0.17715 | -8.44 | -4.2385 | 133 | 14 | 4.05038 | 5.11E-05 | 0.000483 |
| PPCDC | 15 | 0.0602 | rs4886655 | 6 | rs11854704 | NA | 0 | -4.8448 | 67 | 67 | -4.0502 | 5.12E-05 | 0.000483 |
| FAM177A1 | 14 | 0.0443 | rs12100841 | -5.33 | rs17456102 | 0.0069 | -3.67 | -4.75 | 92 | 92 | 4.041 | 5.32E-05 | 0.000498 |
| HARS2 | 5 | 0.0315 | rs801167 | -4.81 | rs778596 | 0.02177 | 3.54 | -4.4118 | 77 | 77 | -4.03038 | 5.57E-05 | 0.000518 |
| RGS6 | 14 | 0.1183 | rs11463 | 5.89 | rs36341 | 0.06614 | -5.56 | -2.4706 | 550 | 550 | 4.0242 | 5.72E-05 | 0.000529 |
| GCAT | 22 | 0.3818 | rs2285178 | 5.895 | rs4820297 | NA | 0 | 5.1667 | 60 | 6 | 4.015 | 5.95E-05 | 0.000546 |
| DDX51 | 12 | 0.0358 | rs11246991 | 5.54 | rs7488356 | NA | 0 | 4.7143 | 87 | 87 | 4.011 | 6.05E-05 | 0.000551 |
| GSTM1 | 1 | 0.4259 | rs4970774 | -5.06 | rs3754446 | 0.08989 | -6.01 | -2.8 | 95 | 32 | 4.00967 | 6.08E-05 | 0.000551 |
| NUB1 | 7 | 0.0579 | rs7805834 | -3.64 | rs394259 | 0.00694 | 3.82 | 2.8333 | 140 | 14 | 4.0051 | 6.20E-05 | 0.000559 |
| ADHFE1 | 8 | 0.4407 | rs2303519 | -4.5 | rs1030420 | 0.30383 | 11.09 | -3.5 | 73 | 7 | -3.9952 | 6.46E-05 | 0.000579 |
| SDHA | 5 | 0.0841 | rs6888378 | 4.46 | rs9312983 | 0.05286 | -5.28 | 2.9805 | 109 | 6 | -3.99315 | 6.52E-05 | 0.00058 |
| FAM160B2 | 8 | 0.0335 | rs6557791 | 3.86 | rs17296501 | NA | 0 | -3.25 | 91 | 3 | -3.9894 | 6.62E-05 | 0.000585 |
| LRP4 | 11 | 0.0423 | rs7128102 | 5.93 | rs2306029 | 0.00266 | 3.44 | 1.3529 | 66 | 66 | -3.9885 | 6.65E-05 | 0.000585 |
| SLC25A11 | 17 | 0.039 | rs3966782 | 7.21 | rs2586532 | -0.0002 | -3.11 | 2.7789 | 95 | 95 | 3.9567 | 7.60E-05 | 0.000662 |
| TGFBRAP1 | 2 | 0.0625 | rs3087523 | 6.46 | rs873738 | 0.01442 | 3.84 | 4 | 157 | 157 | 3.9549 | 7.66E-05 | 0.000662 |
| ISLR2 | 15 | 0.0701 | rs4886868 | 5.48 | rs2304716 | NA | 0 | -2.8889 | 63 | 63 | -3.9519 | 7.75E-05 | 0.000662 |
| GSTM5 | 1 | 0.3355 | rs4970774 | -5.12 | rs3754446 | 0.314 | -10.9 | -2.8 | 89 | 28 | 3.95211 | 7.75E-05 | 0.000662 |
| NUDCD3 | 7 | 0.0538 | rs11760674 | 5.65 | rs1043084 | 0.0833 | 5.76 | 3.9048 | 106 | 2 | 3.9516 | 7.76E-05 | 0.000662 |
| RCN1 | 11 | 0.0676 | rs223051 | -6.22 | rs224633 | 0.08863 | -5.87 | 3.9474 | 117 | 6 | -3.9462 | 7.94E-05 | 0.000673 |
| CYSTM1 | 5 | 0.0933 | rs2074613 | -6.53 | rs1623177 | NA | 0 | -4.1053 | 74 | 74 | 3.93623 | 8.28E-05 | 0.000698 |
| CPT1A | 11 | 0.0486 | rs679596 | 5.76 | rs4460815 | 0.04221 | 4.51 | -1.2298 | 120 | 120 | -3.9308 | 8.47E-05 | 0.00071 |
| TOM1L2 | 17 | 0.0616 | rs752579 | -4.82 | rs7501812 | 0.11 | -6.9 | -3.6111 | 117 | 12 | 3.9252 | 8.67E-05 | 0.000723 |
| NUCB2 | 11 | 0.0907 | rs2051772 | -7.29 | rs739689 | 0.02879 | 4.85 | 3.2094 | 97 | 97 | 3.9076 | 9.32E-05 | 0.000772 |
| CPS1 | 2 | 0.0379 | rs4673553 | 8.35 | rs1509816 | NA | 0 | -0.5161 | 133 | 133 | 3.8878 | 0.0001 | 0.000828 |
| SLC22A23 | 6 | 0.075 | rs9503598 | -3.56 | rs9503598 | 0.03402 | -4.52 | -3.5586 | 226 | 4 | 3.888 | 0.0001 | 0.000828 |
| MKRN2 | 3 | 0.1604 | rs11706408 | 3.6 | rs11710163 | 0.08302 | 5.99 | 3.5484 | 84 | 21 | 3.87741 | 0.00011 | 0.000864 |
| RFT1 | 3 | 0.0815 | rs1080500 | 7.88 | rs2115779 | NA | 0 | -5.6667 | 88 | 88 | -3.82035 | 0.00013 | 0.001077 |
| CTSB | 8 | 0.0918 | rs804280 | -8.18 | rs1293322 | 0.08741 | 6.44 | -3.25 | 145 | 145 | -3.8183 | 0.00013 | 0.001079 |
| MRPL9 | 1 | 0.0425 | rs6587615 | -3.82 | rs1211981 | NA | 0 | -2.6842 | 87 | 87 | 3.78661 | 0.00015 | 0.001226 |
| SYNM | 15 | 0.0777 | rs1670262 | -5.21 | rs1703770 | 0.05186 | -5.47 | 2.65 | 162 | 3 | -3.778 | 0.00016 | 0.001259 |
| RP11-399J13 | 11 | 0.1268 | rs492799 | -4.52 | rs2285346 | NA | 0 | 0.4762 | 98 | 22 | -3.7738 | 0.00016 | 0.001276 |
| PPP2R5C | 14 | 0.295 | rs10431745 | -6.15 | rs736449 | 0.11675 | -7.09 | -2.7931 | 93 | 29 | 3.7727 | 0.00016 | 0.001277 |
| GSTM2 | 1 | 0.2829 | rs4970774 | -5 | rs3754446 | 0.15615 | -8.54 | -2.8 | 89 | 25 | 3.74383 | 0.00018 | 0.001418 |
| COQ5 | 12 | 0.0863 | rs16950287 | -4.5 | rs10774555 | 0.08632 | 6.01 | 3.2444 | 88 | 4 | 3.735 | 0.00019 | 0.001465 |
| TRIM65 | 17 | 0.1353 | rs9894383 | 5.73 | rs10221244 | NA | 0 | 5.2778 | 59 | 4 | 3.7338 | 0.00019 | 0.001465 |
| TMEM160 | 19 | 0.09 | rs3810291 | -15.22 | rs311384 | NA | 0 | 0.3684 | 36 | 7 | 3.73 | 0.00019 | 0.001473 |
| VAV3 | 1 | 0.1105 | rs1104781 | 5.11 | rs4914946 | NA | 0 | -3.1765 | 343 | 343 | 3.72815 | 0.00019 | 0.00148 |
| NUP210 | 3 | 0.0871 | rs580438 | 6.33 | rs9874760 | 0.03648 | 4.05 | 4.6667 | 148 | 148 | 3.72466 | 0.0002 | 0.001495 |
| C14orf159 | 14 | 0.5009 | rs4904744 | -7.42 | rs4900072 | 0.48692 | -13.62 | -3.6962 | 107 | 3 | 3.7186 | 2.00E-04 | 0.00151 |
| FDX1L | 19 | 0.1317 | rs2278442 | 4.33 | rs12720356 | 0.0736 | -5.55 | 4.2333 | 84 | 4 | -3.719 | 2.00E-04 | 0.00151 |
| PGP | 16 | 0.0414 | rs3160 | 3.83 | rs26848 | 0.03644 | 3.98 | 2.8889 | 56 | 8 | 3.7089 | 0.00021 | 0.001554 |
| MYO6 | 6 | 0.2129 | rs3765140 | -4.22 | rs1280054 | 0.26162 | -10.5 | -3.7647 | 143 | 15 | 3.709 | 0.00021 | 0.001554 |
| PPP5C | 19 | 0.0411 | rs3745787 | 4.89 | rs877589 | -0.0014 | 3.22 | 3.7895 | 108 | 108 | 3.701 | 0.00022 | 0.001598 |
| SYTL2 | 11 | 0.1471 | rs11606688 | -5.86 | rs494119 | NA | 0 | 3.75 | 141 | 141 | 3.6978 | 0.00022 | 0.001605 |
| TTC38 | 22 | 0.2819 | rs4253755 | 5.038 | rs4253650 | NA | 0 | 0.8508 | 86 | 8 | -3.693 | 0.00022 | 0.001626 |
| STMN4 | 8 | 0.0627 | rs17446091 | 6.15 | rs2322509 | NA | 0 | -0.4706 | 93 | 12 | -3.692 | 0.00022 | 0.001632 |
| CAST | 5 | 0.0891 | rs3822683 | -6.9 | rs152005 | 0.06574 | 5.45 | -3.625 | 172 | 8 | -3.67802 | 0.00024 | 0.001711 |
| KIAA1324 | 1 | 0.0732 | rs17584208 | 6.41 | rs12239854 | 0.03926 | -5.35 | -3.3913 | 125 | 14 | 3.67395 | 0.00024 | 0.001732 |
| RAB7L1 | 1 | 0.0678 | rs708727 | -6.53 | rs16856139 | NA | 0 | -0.878 | 92 | 23 | -3.64715 | 0.00027 | 0.001908 |
| ETFB | 19 | 0.0727 | rs2062655 | 6.39 | rs3786625 | 0.03732 | 4.8 | 3.5 | 106 | 5 | 3.646 | 0.00027 | 0.001908 |
| TLDC1 | 16 | 0.16 | rs408022 | 3.39 | rs422918 | 0.06093 | -5.86 | -1 | 270 | 270 | 3.6388 | 0.00027 | 0.001956 |
| LGALS3 | 14 | 0.0955 | rs873061 | -5.05 | rs1952438 | NA | 0 | 1.0435 | 60 | 5 | 3.6365 | 0.00028 | 0.001961 |
| PDPR | 16 | 0.0796 | rs4985407 | -8.17 | rs4985520 | 0.00245 | 2.66 | 0.3882 | 15 | 15 | -3.6186 | 0.0003 | 0.002082 |
| DAGLB | 7 | 0.112 | rs7784465 | 6.56 | rs836547 | -0.0025 | -3.07 | -0.1765 | 98 | 98 | -3.6184 | 0.0003 | 0.002082 |
| TDRKH | 1 | 0.0491 | rs6587615 | -3.82 | rs1054475 | 0.0214 | -4.25 | -2.4444 | 95 | 95 | 3.59935 | 0.00032 | 0.002233 |
| FAM171A1 | 10 | 0.251 | rs10906857 | 5 | rs3814165 | 0.18934 | -8.6 | -4.087 | 198 | 198 | 3.5968 | 0.00032 | 0.002243 |
| LANCL1 | 2 | 0.0264 | rs1458281 | 4.24 | rs2287420 | 0.04317 | 4.23 | 3.5882 | 91 | 1 | 3.5882 | 0.00033 | 0.002309 |
| ETFA | 15 | 0.0561 | rs2959863 | 4.43 | rs2456046 | 0.04098 | -5.04 | -2.8235 | 116 | 116 | 3.586 | 0.00034 | 0.002318 |
| UFL1 | 6 | 0.0343 | rs2983897 | 3.76 | rs2983897 | 0.04571 | 4.63 | 3.7619 | 79 | 79 | 3.57 | 0.00036 | 0.002451 |
| AP3D1 | 19 | 0.0502 | rs12462556 | 3.96 | rs2072304 | 0.0278 | -4.07 | 3.5652 | 72 | 1 | -3.565 | 0.00036 | 0.002488 |
| CHMP1A | 16 | 0.0468 | rs101151 | -5.77 | rs164749 | 0.00931 | -4 | 2.6875 | 87 | 87 | -3.5597 | 0.00037 | 0.002524 |
| PSMA8 | 18 | 0.0325 | rs11661836 | 4.53 | rs12960119 | NA | 0 | -3.806 | 48 | 48 | -3.552 | 0.00038 | 0.002593 |
| TBCE | 1 | 0.4258 | rs10925942 | 5.23 | rs4659838 | 0.02415 | 4.77 | -4.3333 | 95 | 10 | -3.54817 | 0.00039 | 0.002609 |
| FARP1 | 13 | 0.1166 | rs7334078 | -6.37 | rs8002818 | NA | 0 | 0.0286 | 353 | 353 | 3.5471 | 0.00039 | 0.002609 |
| LACTB2 | 8 | 0.0855 | rs441890 | -3.81 | rs12681420 | 0.08932 | 6.6 | 3.2941 | 66 | 7 | 3.5363 | 0.00041 | 0.00271 |
| EPM2A | 6 | 0.1535 | rs702322 | 3.33 | rs702322 | 0.03356 | -3.9 | 3.3333 | 74 | 4 | -3.531 | 0.00041 | 0.002751 |
| THOP1 | 19 | 0.0247 | rs7343093 | 4.13 | rs12608998 | 0.01229 | 3.4 | -2.9524 | 101 | 101 | -3.53 | 0.00042 | 0.002752 |
| CSDC2 | 22 | 0.1531 | rs738140 | -7.105 | rs5758365 | NA | 0 | 3.7727 | 42 | 42 | 3.527 | 0.00042 | 0.002772 |
| CARM1 | 19 | 0.0305 | rs11670365 | 4.17 | rs11085749 | 0.02938 | 4.46 | 2.9444 | 70 | 5 | 3.518 | 0.00044 | 0.002858 |
| ADD1 | 4 | 0.0385 | rs2488815 | -4.88 | rs2071695 | 0.0133 | -3.74 | 1.273 | 108 | 108 | -3.5063 | 0.00045 | 0.002962 |
| SERPINF1 | 17 | 0.0741 | rs8067195 | 4.13 | rs1136287 | 0.0296 | -3.54 | -0.0556 | 104 | 104 | 3.4956 | 0.00047 | 0.003072 |
| SELENBP1 | 1 | 0.2263 | rs2769265 | 4.77 | rs17564336 | 0.13463 | -7.35 | -3.2105 | 58 | 5 | 3.4872 | 0.00049 | 0.003156 |
| LARS | 5 | 0.044 | rs2063002 | -3.74 | rs12188581 | 0.06958 | -5.65 | -3.4737 | 87 | 4 | 3.48457 | 0.00049 | 0.003166 |
| C6orf57 | 6 | 0.0254 | rs2691505 | 3.78 | rs6455371 | 0.01537 | -3.7 | 3.6667 | 76 | 76 | -3.484 | 0.00049 | 0.003166 |
| PDDC1 | 11 | 0.0416 | rs11246328 | -8.38 | rs4073591 | NA | 0 | -2.0796 | 104 | 14 | 3.4747 | 0.00051 | 0.00326 |
| AVEN | 15 | 0.0622 | rs8042524 | 3.55 | rs12906584 | NA | 0 | 2.1035 | 196 | 196 | -3.4722 | 0.00052 | 0.003278 |
| AUH | 9 | 0.0917 | rs7869771 | -7.37 | rs4743820 | 0.0872 | 6.94 | -3.2105 | 130 | 8 | -3.4575 | 0.00055 | 0.003447 |
| CENPV | 17 | 0.0329 | rs12946619 | -5.54 | rs17779879 | 0.0646 | 5.08 | -2.1154 | 73 | 3 | -3.4537 | 0.00055 | 0.003482 |
| RASAL1 | 12 | 0.0522 | rs7970839 | 4.65 | rs1902955 | 0.00298 | -2.82 | -2.4444 | 87 | 87 | -3.442 | 0.00058 | 0.003611 |
| C2CD2L | 11 | 0.0791 | rs1003081 | 7.56 | rs4938626 | 0.06954 | -5.64 | -3.4236 | 101 | 2 | 3.4233 | 0.00062 | 0.003864 |
| GGPS1 | 1 | 0.0297 | rs2291687 | -4.56 | rs12035746 | 0.04441 | 4.42 | -3.4118 | 52 | 1 | -3.41176 | 0.00065 | 0.004009 |
| POR | 7 | 0.3728 | rs3823882 | 5.74 | rs10954732 | 0.05785 | 5.05 | 4.9444 | 73 | 14 | 3.3991 | 0.00068 | 0.004184 |
| FXN | 9 | 0.168 | rs10869918 | 2.59 | rs2498432 | 0.04752 | 5.73 | -2.541 | 115 | 5 | -3.385 | 0.00071 | 0.004388 |
| APIP | 11 | 0.2729 | rs2762954 | 4.32 | rs7929679 | NA | 0 | -1.2941 | 150 | 17 | 3.3824 | 0.00072 | 0.004412 |
| KIAA1161 | 9 | 0.1963 | rs4879788 | -4.56 | rs10972032 | NA | 0 | 0.5 | 74 | 10 | -3.3518 | 0.0008 | 0.004906 |
| TPPP3 | 16 | 0.0586 | rs8052687 | 4.3 | rs3868143 | 0.05622 | -5.01 | 2.4848 | 50 | 3 | -3.3494 | 0.00081 | 0.004928 |
| OXNAD1 | 3 | 0.2406 | rs712873 | 3.14 | rs4685319 | NA | 0 | 1.3333 | 179 | 19 | 3.33452 | 0.00085 | 0.005174 |
| TREX1 | 3 | 0.0435 | rs6442117 | -3.47 | rs4558783 | NA | 0 | -2.677 | 62 | 3 | 3.3212 | 0.0009 | 0.005401 |
| RUFY1 | 5 | 0.0524 | rs4701140 | -4.28 | rs4563584 | 0.00477 | 3.37 | 2.5098 | 93 | 93 | 3.32049 | 0.0009 | 0.005401 |
| AKR7A3 | 1 | 0.0695 | rs3762396 | 4.29 | rs12133034 | 0.02911 | -4.66 | -1.3793 | 117 | 117 | 3.31813 | 0.00091 | 0.005421 |
| DCXR | 17 | 0.0637 | rs11658335 | 5.94 | rs34319293 | NA | 0 | -0.11 | 61 | 61 | -3.3135 | 0.00092 | 0.005488 |
| SARM1 | 17 | 0.2463 | rs4795430 | 4.45 | rs739439 | 0.162 | 7.99 | -3.0476 | 79 | 79 | -3.3099 | 0.00093 | 0.005537 |
| TRPT1 | 11 | 0.0868 | rs34882006 | -8.47 | rs7112960 | NA | 0 | -3.7778 | 68 | 8 | 3.3045 | 0.00095 | 0.00562 |
| DDAH1 | 1 | 0.0748 | rs582145 | 4.88 | rs233109 | 0.03045 | 4.71 | -2.4444 | 255 | 255 | -3.30273 | 0.00096 | 0.005633 |
| CAMLG | 5 | 0.2973 | rs2108665 | 3.29 | rs11747528 | NA | 0 | -0.9506 | 29 | 4 | -3.28692 | 0.00101 | 0.00592 |
| NAE1 | 16 | 0.0458 | rs543419 | 3.84 | rs3026082 | 0.00741 | 3.92 | 2.0448 | 77 | 77 | 3.2835 | 0.00103 | 0.005989 |
| SIDT1 | 3 | 0.3158 | rs13092825 | -3.18 | rs6768117 | NA | 0 | -1.1177 | 114 | 18 | -3.28271 | 0.00103 | 0.005989 |
| PARK2 | 6 | 0.1086 | rs13191362 | -9.44 | rs9346868 | -0.002 | -3.72 | -0.9412 | 946 | 946 | -3.269 | 0.00108 | 0.006254 |
| ACSF3 | 16 | 0.3257 | rs2270416 | -5.3 | rs4785602 | 0.0123 | 4.93 | 2.6316 | 77 | 33 | 3.2615 | 0.00111 | 0.006402 |
| ARHGEF25 | 12 | 0.0626 | rs7313599 | -4.89 | rs697221 | NA | 0 | 1.76 | 84 | 2 | 3.259 | 0.00112 | 0.006434 |
| CSPG4 | 15 | 0.0292 | rs2009508 | 4.16 | rs4503758 | -0.0025 | -3.31 | 4.1053 | 60 | 60 | -3.2556 | 0.00113 | 0.006466 |
| RCHY1 | 4 | 0.0812 | rs1478174 | 3.5 | rs12501934 | NA | 0 | 3.42 | 85 | 85 | -3.25 | 0.00115 | 0.006555 |
| FSCN1 | 7 | 0.038 | rs6463489 | 6.04 | rs4320462 | 0.00962 | 3.45 | 2 | 76 | 76 | 3.2482 | 0.00116 | 0.006586 |
| MTRF1L | 6 | 0.1152 | rs2185027 | 7.5 | rs9371645 | NA | 0 | 1 | 146 | 3 | -3.24 | 0.00119 | 0.006729 |
| ABCC8 | 11 | 0.0561 | rs2051772 | -7.29 | rs1330 | NA | 0 | -4.6471 | 221 | 1 | -3.2382 | 0.0012 | 0.006759 |
| TP53I3 | 2 | 0.1581 | rs2136264 | 4.28 | rs12616250 | NA | 0 | -1.5417 | 77 | 3 | -3.2376 | 0.00121 | 0.006789 |
| GSTM4 | 1 | 0.0771 | rs4970774 | -5.03 | rs12046010 | NA | 0 | 4.4546 | 83 | 83 | 3.23466 | 0.00122 | 0.006819 |
| CACNA2D3 | 3 | 0.1005 | rs1492001 | -3.84 | rs750379 | 0.02192 | 4.22 | 1.5 | 690 | 690 | 3.22352 | 0.00127 | 0.007071 |
| LRRC8A | 9 | 0.0667 | rs10988152 | -3.32 | rs10988151 | 0.04451 | -4.77 | -3.1053 | 73 | 4 | 3.2151 | 0.0013 | 0.00721 |
| GPRIN3 | 4 | 0.1482 | rs4693992 | -4.22 | rs2005104 | 0.09075 | 6.02 | 2.889 | 113 | 5 | 3.2072 | 0.00134 | 0.007403 |
| GOSR2 | 17 | 0.0632 | rs8080126 | 4 | rs17676978 | 0.01 | -4.85 | 3.0741 | 86 | 86 | -3.1942 | 0.0014 | 0.007705 |
| GALC | 14 | 0.3074 | rs8009710 | 6.7 | rs416542 | 0.23074 | -9.8 | -0.4706 | 150 | 44 | -3.1803 | 0.00147 | 0.00806 |
| UQCR10 | 22 | 0.1109 | rs7290420 | 3.571 | rs17711508 | 0.10932 | -7.13 | 3.122 | 84 | 4 | -3.167 | 0.00154 | 0.008412 |
| TXNDC5 | 6 | 0.0554 | rs1043784 | 4.22 | rs9505293 | 0.05231 | 4.7 | 3.7143 | 165 | 4 | 3.162 | 0.00156 | 0.008489 |
| RPA2 | 1 | 0.0267 | rs17257252 | -3.1 | rs17257252 | 0.04403 | 4.41 | -3.1 | 69 | 2 | -3.15622 | 0.0016 | 0.008674 |
| ABHD12 | 20 | 0.2138 | rs8122855 | 7.33 | rs746748 | 0.08949 | -6.03 | -3.3429 | 137 | 5 | 3.1521 | 0.00162 | 0.008749 |
| PANX1 | 11 | 0.0839 | rs11607757 | 3.63 | rs2460055 | NA | 0 | -0.5882 | 84 | 84 | -3.1487 | 0.00164 | 0.008759 |
| SPAG7 | 17 | 0.1162 | rs3966782 | 7.21 | rs8078173 | NA | 0 | 4.8333 | 92 | 2 | 3.148 | 0.00164 | 0.008759 |
| 2-Mar | 1 | 0.0566 | rs2738755 | -4.83 | rs6697965 | 0.07526 | -5.75 | 2 | 132 | 13 | -3.14852 | 0.00164 | 0.008759 |
| AMPD3 | 11 | 0.1611 | rs10219205 | -3.89 | rs11604833 | 0.12333 | 6.91 | 2.4362 | 171 | 171 | 3.1459 | 0.00166 | 0.0088 |
| PTGR1 | 9 | 0.1912 | rs2273786 | 4.94 | rs3739704 | 0.14251 | 7.48 | -3.1667 | 111 | 2 | -3.1458 | 0.00166 | 0.0088 |
| ARNT2 | 15 | 0.0603 | rs715724 | -7.65 | rs3901896 | 0.01431 | 4.49 | -2.6667 | 175 | 175 | -3.137 | 0.00171 | 0.009032 |
| GLO1 | 6 | 0.06 | rs2490026 | -4 | rs1781735 | 0.04584 | -4.85 | -3.0588 | 106 | 3 | 3.102 | 0.00192 | 0.010105 |
| LNPEP | 5 | 0.0426 | rs4092590 | -3.96 | rs18059 | 0.0219 | 3.96 | -2.5882 | 123 | 123 | -3.0966 | 0.00196 | 0.010278 |
| AK4 | 1 | 0.1051 | rs552327 | -4.24 | rs4511159 | 0.09473 | 6.44 | 2.9444 | 126 | 4 | 3.08326 | 0.00205 | 0.010711 |
| FBP1 | 9 | 0.1072 | rs10117499 | -3.77 | rs7860616 | NA | 0 | -2.6008 | 112 | 112 | -3.0782 | 0.00208 | 0.010828 |
| VSTM2B | 19 | 0.1008 | rs1529721 | 3.42 | rs1079335 | NA | 0 | 0.1765 | 158 | 158 | -3.075 | 0.00211 | 0.010945 |
| ABCB9 | 12 | 0.0532 | rs11060406 | -4.53 | rs11060942 | 0.05161 | 4.64 | -3.524 | 50 | 2 | -3.071 | 0.00214 | 0.011061 |
| LRPPRC | 2 | 0.1059 | rs4953042 | -4.35 | rs7568481 | 0.05651 | 5.72 | -0.8889 | 177 | 14 | -3.0691 | 0.00215 | 0.011073 |
| PSMB4 | 1 | 0.2373 | rs2769265 | 4.77 | rs4603 | 0.45892 | -13.13 | 3.5417 | 52 | 13 | -3.06315 | 0.00219 | 0.011238 |
| PIK3CD | 1 | 0.059 | rs1135427 | 4 | rs6660810 | NA | 0 | -2.0952 | 87 | 87 | 3.0587 | 0.00222 | 0.011352 |
| B4GALNT1 | 12 | 0.0273 | rs7313599 | -4.89 | rs2127318 | NA | 0 | 1.92 | 88 | 88 | 3.058 | 0.00223 | 0.011363 |
| NFXL1 | 4 | 0.1525 | rs10938509 | -3.19 | rs4473628 | NA | 0 | -1.389 | 79 | 9 | -3.0516 | 0.00228 | 0.011577 |
| NT5DC3 | 12 | 0.2702 | rs728167 | 4 | rs7314150 | 0.07411 | 6.85 | 3.25 | 251 | 251 | 3.05 | 0.00229 | 0.011587 |
| AAGAB | 15 | 0.0346 | rs3825977 | -5.27 | rs17213990 | NA | 0 | -1.4615 | 120 | 120 | 3.0453 | 0.00232 | 0.011697 |
| C5 | 9 | 0.0643 | rs3761847 | -3.06 | rs13291973 | NA | 0 | 0.1935 | 119 | 4 | -3.0427 | 0.00235 | 0.011807 |
| NLRX1 | 11 | 0.1256 | rs1185460 | 7.43 | rs4938637 | 0.05939 | 5.25 | -4.9118 | 85 | 6 | -3.0236 | 0.0025 | 0.012474 |
| IDUA | 4 | 0.1795 | rs6855019 | -3.49 | rs2279181 | NA | 0 | -1.166 | 110 | 4 | -3.0232 | 0.0025 | 0.012474 |
| WDFY1 | 2 | 0.0476 | rs10196123 | 2.59 | rs893360 | 0.02502 | -4.16 | 2.4444 | 129 | 129 | -3.0076 | 0.00263 | 0.013077 |
| KIAA1468 | 18 | 0.055 | rs17069628 | 3.76 | rs9320002 | 0.0749 | -5.45 | 3 | 134 | 2 | -3 | 0.0027 | 0.013379 |
| C6orf211 | 6 | 0.0492 | rs7765741 | 4.9 | rs7771156 | 0.05496 | -4.69 | 4 | 97 | 97 | -2.982 | 0.00286 | 0.014124 |
| DEPDC5 | 22 | 0.0409 | rs2006648 | 5.286 | rs3213524 | 0.01366 | -4.1 | 3.0357 | 113 | 113 | -2.981 | 0.00287 | 0.014125 |
| GFRA1 | 10 | 0.1707 | rs180620 | -3.09 | rs7087751 | NA | 0 | -0.1667 | 250 | 19 | 2.9781 | 0.0029 | 0.014224 |
| GMPR | 6 | 0.0514 | rs7763322 | -3.61 | rs7763395 | NA | 0 | -1.579 | 110 | 110 | -2.969 | 0.00298 | 0.014566 |
| FAM118B | 11 | 0.0591 | rs649142 | 3.33 | rs543969 | NA | 0 | -0.0952 | 173 | 173 | -2.9689 | 0.00299 | 0.014566 |
| NENF | 1 | 0.0421 | rs1021379 | -4.15 | rs11119918 | NA | 0 | 2.0455 | 104 | 4 | -2.95843 | 0.00309 | 0.015003 |
| SLC17A6 | 11 | 0.0633 | rs10833748 | -3.61 | rs11026523 | 0.04246 | -4.4 | -2.8095 | 96 | 3 | 2.9545 | 0.00313 | 0.015146 |
| CRK | 17 | 0.0352 | rs11651209 | -3.47 | rs16946807 | -0.0018 | 3.39 | -2.5882 | 92 | 92 | -2.9489 | 0.00319 | 0.015385 |
| SGTB | 5 | 0.0923 | rs754784 | 3.59 | rs1549192 | 0.08505 | -6.08 | 3 | 122 | 3 | -2.93411 | 0.00335 | 0.016102 |
| SIRT3 | 11 | 0.0488 | rs1045288 | -4.25 | rs7930823 | -0.0022 | -3.41 | -3.7895 | 106 | 106 | 2.911 | 0.0036 | 0.017247 |
| RPAP1 | 15 | 0.057 | rs10775122 | 6.45 | rs35826725 | NA | 0 | -1.2003 | 79 | 79 | -2.9073 | 0.00365 | 0.017371 |
| GALT | 9 | 0.0916 | rs10758268 | -5.29 | rs4395980 | NA | 0 | -0.55 | 88 | 88 | 2.9065 | 0.00365 | 0.017371 |
| PNMAL2 | 19 | 0.055 | rs3745787 | 4.89 | rs892106 | 0.01712 | -4.71 | 1.7274 | 89 | 89 | -2.899 | 0.00374 | 0.017661 |
| PACS1 | 11 | 0.0378 | rs524859 | -6.28 | rs9326370 | 0.02041 | 4 | -5.7059 | 131 | 19 | -2.8982 | 0.00375 | 0.017661 |
| C5orf51 | 5 | 0.0443 | rs276278 | -2.75 | rs34663371 | 0.00366 | 3.26 | -2.2962 | 50 | 8 | -2.8981 | 0.00375 | 0.017661 |
| SAMD4A | 14 | 0.3041 | rs2183081 | -3.78 | rs10129440 | NA | 0 | -2.75 | 219 | 8 | 2.8978 | 0.00376 | 0.017661 |
| PMPCB | 7 | 0.062 | rs17680523 | 3.26 | rs17680523 | 0.03301 | -3.93 | 3.2581 | 26 | 17 | -2.8815 | 0.00396 | 0.01854 |
| PPAT | 4 | 0.0893 | rs14211 | -2.96 | rs6836118 | 0.09587 | 6.22 | -2.822 | 103 | 103 | -2.8768 | 0.00402 | 0.01876 |
| ARL2BP | 16 | 0.1908 | rs11864646 | 3.03 | rs7198865 | 0.08796 | -5.88 | 2.8846 | 76 | 2 | -2.8745 | 0.00405 | 0.018778 |
| AKR7A2 | 1 | 0.1325 | rs710875 | 3.56 | rs10158374 | NA | 0 | -1.9231 | 124 | 4 | 2.87425 | 0.00405 | 0.018778 |
| RP4-583P15 | 20 | 0.105 | rs6011118 | -4.67 | rs2872810 | NA | 0 | -2.85 | 89 | 2 | 2.8674 | 0.00414 | 0.019134 |
| PTPN11 | 12 | 0.0303 | rs11066301 | -7.12 | rs10850031 | 0.02004 | 4.05 | 4.1667 | 58 | 58 | 2.854 | 0.00432 | 0.019839 |
| DHODH | 16 | 0.4181 | rs4788454 | -7.59 | rs7184117 | 0.24528 | -9.75 | -3.6842 | 126 | 23 | 2.854 | 0.00432 | 0.019839 |
| RNF141 | 11 | 0.1627 | rs10840441 | -3.9 | rs1993819 | 0.1116 | 7.62 | -3.8235 | 145 | 26 | -2.8425 | 0.00448 | 0.020508 |
| NDUFS1 | 2 | 0.0206 | rs13028887 | -3.95 | rs818018 | 0.02175 | 3.6 | 1.6111 | 91 | 7 | 2.8405 | 0.0045 | 0.020535 |
| ZADH2 | 18 | 0.2719 | rs9962947 | -4.06 | rs7230037 | 0.221 | -9.33 | 2.849 | 136 | 4 | -2.834 | 0.00459 | 0.020879 |
| LRRC16A | 6 | 0.1343 | rs9366627 | -5.83 | rs1076668 | 0.02656 | 5.39 | 2.3809 | 332 | 20 | 2.823 | 0.00476 | 0.021585 |
| ENDOG | 9 | 0.0462 | rs10988152 | -3.32 | rs3750325 | NA | 0 | -0.4681 | 58 | 5 | 2.8115 | 0.00493 | 0.022285 |
| VPS36 | 13 | 0.1508 | rs9536046 | -2.72 | rs12430144 | 0.05493 | -5.47 | -2.65 | 49 | 3 | 2.8098 | 0.00496 | 0.022351 |
| CBR1 | 21 | 0.2142 | rs1005696 | -2.88 | rs1005696 | 0.0993 | 6.33 | -2.8824 | 115 | 4 | -2.8073 | 0.005 | 0.022461 |
| TMTC4 | 13 | 0.1308 | rs2786951 | -4.89 | rs837290 | NA | 0 | -2.2632 | 124 | 124 | 2.8043 | 0.00504 | 0.02257 |
| NUAK1 | 12 | 0.0655 | rs1215600 | 3 | rs17038085 | 0.05904 | 4.85 | -2.8182 | 185 | 3 | -2.802 | 0.00507 | 0.022634 |
| ADCK1 | 14 | 0.2464 | rs17752950 | -4.04 | rs4899673 | NA | 0 | 2.3529 | 199 | 9 | -2.7989 | 0.00513 | 0.022761 |
| TRAP1 | 16 | 0.1971 | rs9930893 | -7.59 | rs2791 | 0.09944 | -7.91 | 0.0972 | 62 | 23 | -2.799 | 0.00513 | 0.022761 |
| SFXN3 | 10 | 0.407 | rs11190766 | 5.35 | rs7099531 | 0.08011 | -6.9 | -0.7917 | 101 | 24 | 2.7967 | 0.00516 | 0.022824 |
| STAU1 | 20 | 0.0409 | rs10485609 | -6.9 | rs6066968 | 0.03288 | 3.81 | 2.1176 | 92 | 92 | 2.7918 | 0.00524 | 0.023107 |
| FAM221A | 7 | 0.105 | rs227932 | -3.61 | rs227932 | NA | 0 | -3.6071 | 92 | 5 | 2.7906 | 0.00526 | 0.023125 |
| SIRPA | 20 | 0.5504 | rs6045210 | 3.94 | rs6075340 | 0.55156 | -14.41 | 2.7778 | 162 | 1 | -2.7778 | 0.00547 | 0.023946 |
| PEPD | 19 | 0.2408 | rs11882409 | 6.32 | rs3556 | 0.27191 | 10.45 | 1.2 | 157 | 7 | 2.777 | 0.00548 | 0.023946 |
| CYBRD1 | 2 | 0.2201 | rs3821083 | -6.15 | rs7590275 | NA | 0 | -1.8333 | 113 | 15 | 2.7721 | 0.00557 | 0.024177 |
| EEFSEC | 3 | 0.039 | rs9855048 | 3.91 | rs2335772 | NA | 0 | 0 | 166 | 4 | 2.7723 | 0.00557 | 0.024177 |
| YOD1 | 1 | 0.0391 | rs2075863 | -3.29 | rs4844369 | NA | 0 | -0.9091 | 89 | 4 | -2.77085 | 0.00559 | 0.024177 |
| FAHD2B | 2 | 0.1766 | rs10496325 | 3.18 | rs1257029 | NA | 0 | -1.1539 | 18 | 6 | -2.7701 | 0.0056 | 0.024177 |
| CRYZ | 1 | 0.5587 | rs11210489 | 6.88 | rs17552114 | 0.08435 | -7.61 | -0.0488 | 104 | 36 | -2.7652 | 0.00569 | 0.024492 |
| CYB5R3 | 22 | 0.0931 | rs7287384 | -4.128 | rs137087 | 0.09538 | -6.84 | -2.4009 | 98 | 98 | 2.76 | 0.00578 | 0.024806 |
| ANXA3 | 4 | 0.1343 | rs4466067 | 3.56 | rs1472365 | 0.01277 | 3.64 | 3.105 | 149 | 149 | 2.7547 | 0.00587 | 0.025108 |
| GLRX5 | 14 | 0.5065 | rs10484053 | -2.62 | rs10484053 | 0.32212 | -11.09 | -2.6154 | 216 | 216 | 2.7539 | 0.00589 | 0.025108 |
| C3orf33 | 3 | 0.257 | rs117442 | -4.74 | rs7610374 | NA | 0 | 0.9167 | 96 | 96 | -2.75281 | 0.00591 | 0.025108 |
| PLEKHA1 | 10 | 0.0503 | rs10788284 | 2.71 | rs10510110 | 0.03013 | -4.36 | 2.5882 | 181 | 181 | -2.7523 | 0.00592 | 0.025108 |
| EPS8L2 | 11 | 0.2659 | rs4963120 | -7.22 | rs11246211 | NA | 0 | 0.6665 | 109 | 109 | 2.7447 | 0.00606 | 0.025626 |
| DFNA5 | 7 | 0.2006 | rs1476520 | -4 | rs17207104 | NA | 0 | -1.2245 | 137 | 17 | -2.7272 | 0.00639 | 0.026943 |
| ADA | 20 | 0.285 | rs6031643 | -4.94 | rs6031643 | NA | 0 | -4.9412 | 91 | 91 | -2.7258 | 0.00641 | 0.026948 |
| LHFPL2 | 5 | 0.0644 | rs3922654 | 5.67 | rs10942850 | NA | 0 | 2.4706 | 170 | 170 | -2.72435 | 0.00644 | 0.026996 |
| ACP6 | 1 | 0.4304 | rs11587616 | -4 | rs6657449 | NA | 0 | -1.2241 | 123 | 4 | -2.71906 | 0.00655 | 0.027377 |
| GRWD1 | 19 | 0.1796 | rs1643494 | 3.35 | rs1643487 | 0.03332 | -5.42 | 2.7419 | 62 | 6 | -2.717 | 0.00658 | 0.027423 |
| UMPS | 3 | 0.19 | rs3863065 | 3.07 | rs6764025 | NA | 0 | -1.4357 | 148 | 42 | -2.7138 | 0.00665 | 0.027635 |
| BCS1L | 2 | 0.0403 | rs1427445 | 7.12 | rs12991539 | 0.00487 | -3.18 | 0.3155 | 84 | 84 | -2.7116 | 0.0067 | 0.027649 |
| PDLIM4 | 5 | 0.1334 | rs272885 | 4.22 | rs156055 | NA | 0 | -3.7931 | 123 | 9 | 2.7115 | 0.0067 | 0.027649 |
| CTNND1 | 11 | 0.0302 | rs641325 | 3.11 | rs499188 | 0.02887 | -3.73 | 3.0556 | 60 | 60 | -2.7102 | 0.00673 | 0.027649 |
| PDIA3 | 15 | 0.0404 | rs16977798 | -5.6 | rs12443084 | 0.03177 | -3.81 | -0.9263 | 49 | 49 | -2.7098 | 0.00673 | 0.027649 |
| ANPEP | 15 | 0.0595 | rs1256843 | -3.06 | rs1972435 | NA | 0 | 0.7576 | 104 | 4 | -2.7046 | 0.00684 | 0.028021 |
| MX1 | 21 | 0.0575 | rs886451 | 3.05 | rs469066 | 0.04302 | -4.89 | -2 | 159 | 10 | 2.6972 | 0.00699 | 0.028554 |
| LYRM5 | 12 | 0.0577 | rs11047887 | 3 | rs12229161 | NA | 0 | 2.5417 | 102 | 4 | 2.694 | 0.00707 | 0.028799 |
| HAAO | 2 | 0.0554 | rs4953577 | -5.82 | rs6708408 | NA | 0 | -3.7368 | 123 | 8 | 2.6897 | 0.00715 | 0.029043 |
| VWA8 | 13 | 0.177 | rs12868711 | 3.53 | rs9594643 | 0.14767 | -7.88 | 3.0526 | 166 | 9 | -2.6877 | 0.00719 | 0.029124 |
| CYP7B1 | 8 | 0.0334 | rs4737675 | 4.48 | rs13276064 | NA | 0 | 0.7778 | 143 | 143 | 2.6848 | 0.00726 | 0.029325 |
| MRGPRF | 11 | 0.2702 | rs7940105 | 5.6 | rs10896378 | NA | 0 | -3.65 | 93 | 5 | -2.6822 | 0.00731 | 0.029444 |
| TGOLN2 | 2 | 0.0536 | rs3770102 | -3.16 | rs4247303 | 0.09149 | -6.21 | 2.6471 | 112 | 6 | -2.668 | 0.00763 | 0.030648 |
| MMS19 | 10 | 0.0681 | rs928603 | -4.32 | rs10882950 | 0.02954 | 4.32 | 3.0556 | 106 | 10 | 2.6534 | 0.00797 | 0.031916 |
| RNPEP | 1 | 0.2395 | rs2250377 | 12.83 | rs16849483 | 0.179 | -8.31 | 0.3684 | 96 | 96 | -2.65261 | 0.00799 | 0.031916 |
| DGUOK | 2 | 0.2739 | rs4852983 | 3.22 | rs1653259 | 0.0002 | -3.76 | 0.5676 | 126 | 34 | -2.633 | 0.00846 | 0.0337 |
| HSD17B4 | 5 | 0.1486 | rs1045241 | 3.83 | rs32665 | 0.15846 | -8.44 | 2.4118 | 99 | 5 | -2.62024 | 0.00879 | 0.034918 |
| YEATS4 | 12 | 0.0784 | rs317646 | 7.37 | rs7300266 | NA | 0 | -5.1765 | 115 | 17 | 2.609 | 0.00909 | 0.03601 |
| AAMDC | 11 | 0.2678 | rs4944176 | 2.86 | rs3819211 | 0.37575 | -12.16 | 2.6786 | 85 | 29 | -2.6042 | 0.00921 | 0.036386 |
| MRS2 | 6 | 0.2599 | rs7762901 | -5.05 | rs3846829 | NA | 0 | 4.2174 | 141 | 141 | 2.601 | 0.0093 | 0.03658 |
| GRID1 | 10 | 0.1458 | rs7899106 | 8.95 | rs7075405 | 0.07666 | -5.87 | 3.1639 | 459 | 459 | -2.6005 | 0.00931 | 0.03658 |
| SYNE1 | 6 | 0.0829 | rs12201313 | 4.22 | rs2695253 | 0.01092 | -4.26 | -3.4118 | 466 | 466 | 2.592 | 0.00953 | 0.037343 |
| MGARP | 4 | 0.2144 | rs12646442 | -3.83 | rs4597768 | NA | 0 | 3.333 | 69 | 69 | 2.5893 | 0.00962 | 0.037594 |
| TMEM25 | 11 | 0.0833 | rs2277295 | -3.58 | rs1939950 | NA | 0 | -0.3385 | 67 | 67 | -2.5882 | 0.00965 | 0.037609 |
| CARS2 | 13 | 0.2787 | rs7989346 | 3.42 | rs7337089 | 0.13196 | -8.25 | -2.2273 | 141 | 24 | 2.584 | 0.00976 | 0.037935 |
| PGS1 | 17 | 0.1001 | rs17561950 | -3.65 | rs7212707 | NA | 0 | -0.1333 | 140 | 6 | 2.5822 | 0.00982 | 0.038066 |
| GSTP1 | 11 | 0.1171 | rs11604662 | -3.45 | rs7941648 | 0.18313 | -8.99 | -2.8235 | 76 | 4 | 2.5725 | 0.0101 | 0.039046 |
| NT5C3A | 7 | 0.0733 | rs13311608 | 4.88 | rs3915352 | 0.02798 | 4.34 | 2.3636 | 101 | 19 | 2.5652 | 0.0103 | 0.039713 |
| PPID | 4 | 0.7943 | rs11100197 | 4.35 | rs17843966 | 0.29978 | 10.65 | -2.09 | 29 | 29 | -2.5586 | 0.0105 | 0.040376 |
| MAP4K5 | 14 | 0.0672 | rs17780143 | -4.47 | rs12433794 | NA | 0 | 0.4118 | 115 | 115 | 2.5528 | 0.0107 | 0.040927 |
| DBT | 1 | 0.0775 | rs3131839 | 3.24 | rs2810424 | 0.10737 | -6.58 | 2.5333 | 90 | 5 | -2.55328 | 0.0107 | 0.040927 |
| EML4 | 2 | 0.0864 | rs1992259 | -3.88 | rs6736913 | 0.00038 | -3.19 | 2.0768 | 104 | 104 | -2.5428 | 0.011 | 0.041963 |
| COX7A2L | 2 | 0.0445 | rs1992259 | -3.88 | rs6544546 | 0.01967 | 4.03 | 3.4706 | 88 | 5 | 2.5407 | 0.0111 | 0.042233 |
| SUCLG1 | 2 | 0.03 | rs4831973 | 3.54 | rs6715601 | NA | 0 | -1.0588 | 63 | 3 | 2.5336 | 0.0113 | 0.042881 |
| TXN | 9 | 0.2065 | rs4135165 | -3.03 | rs1049927 | 0.09752 | -6.54 | 1.88 | 142 | 142 | -2.5305 | 0.0114 | 0.043146 |
| MTAP | 9 | 0.1827 | rs1414242 | 3.06 | rs10757257 | 0.20711 | -8.98 | 2.5294 | 113 | 15 | -2.5244 | 0.0116 | 0.043788 |
| CAPG | 2 | 0.1761 | rs3770102 | -3.15 | rs3770102 | 0.11122 | 6.75 | -3.1464 | 88 | 88 | -2.5202 | 0.0117 | 0.044051 |
| APPL2 | 12 | 0.102 | rs1196836 | -5.12 | rs12301914 | 0.00667 | 3.54 | 3.069 | 136 | 14 | 2.519 | 0.0118 | 0.044196 |
| NECAB2 | 16 | 0.1033 | rs7342695 | -3.19 | rs7342695 | 0.05762 | -5.59 | -3.1875 | 175 | 175 | 2.5188 | 0.0118 | 0.044196 |
| NOS1 | 12 | 0.0465 | rs4492907 | 5.44 | rs10774926 | 0.0167 | -4.33 | -1.1739 | 186 | 5 | 2.513 | 0.012 | 0.044829 |
| GTDC1 | 2 | 0.0835 | rs10496954 | -3.9 | rs7589628 | 0.02618 | -4.14 | 3.3529 | 98 | 98 | -2.5101 | 0.0121 | 0.045086 |
| HINT3 | 6 | 0.0232 | rs10447437 | 3.53 | rs2024583 | 0.01628 | -3.64 | 1.8333 | 61 | 61 | -2.498 | 0.0125 | 0.046456 |
| IST1 | 16 | 0.1674 | rs4788454 | -7.59 | rs7195988 | 0.00485 | -4.14 | 0.7453 | 115 | 23 | 2.4956 | 0.0126 | 0.046707 |
| CTSZ | 20 | 0.0494 | rs149265 | 4 | rs163781 | 0.00291 | 3.55 | -2.1765 | 89 | 89 | -2.4896 | 0.0128 | 0.047327 |
| CLN5 | 13 | 0.0731 | rs17066901 | -2.82 | rs617595 | NA | 0 | -0.2857 | 96 | 3 | -2.482 | 0.0131 | 0.048189 |
| RDH11 | 14 | 0.1634 | rs8005363 | -4 | rs718212 | 0.05084 | 4.65 | 1 | 96 | 5 | 2.4805 | 0.0131 | 0.048189 |
| KIAA1279 | 10 | 0.0906 | rs2429025 | 3.39 | rs2491015 | 0.098 | -6.4 | -2.7778 | 93 | 4 | 2.4795 | 0.0132 | 0.048311 |
| SLC44A2 | 19 | 0.0264 | rs12974306 | -4.31 | rs11085744 | 0.01507 | 4.07 | 4.0556 | 85 | 85 | 2.478 | 0.0132 | 0.048311 |
| SRSF9 | 12 | 0.2644 | rs17431446 | -3.87 | rs11065126 | 0.1216 | -7.06 | -2.6464 | 82 | 82 | 2.472 | 0.0134 | 0.048918 |
| HSQ, refers to heritability of the protein using common variants. NSNP, indicates the total number of SNPs within 200kb window (100kb up and downstream) of the gene. NWGT, refers to the total number of SNPs that contribute to the weight of the protein. | | | | | | | | | | | | | |

| Table S7. The PWAS of WHRadjBMI integrating the WHRadjBMI GWAS (N=694,649) with ROS/MAP human brain proteomic and genetic data (N=376) using FUSION. | | | | | | | | | | | | | |
| --- | --- | --- | --- | --- | --- | --- | --- | --- | --- | --- | --- | --- | --- |
| **Gene** | **CHR** | **HSQ** | **BEST.GWAS.ID** | **BEST.GWAS.Z** | **pQTL.ID** | **pQTL.R^2^** | **pQTL.Z** | **pQTL.GWAS.Z** | **NSNP** | **NWGT** | **PWAS.Z** | **PWAS.P** | **PWAS.FDR.Q** |
| CCDC92 | 12 | 0.0507 | rs863750 | -21.94 | rs12309481 | 0.0581 | -5.61 | -14.0526 | 83 | 83 | 21.53 | 8.27E-103 | 1.20E-99 |
| TMEM175 | 4 | 0.0721 | rs4690220 | -9.48 | rs1078139 | NA | 0 | -1.9 | 104 | 104 | -10.29 | 8.15E-25 | 5.90E-22 |
| VASN | 16 | 0.0243 | rs886860 | 10.89 | rs444985 | NA | 0 | 1.0952 | 65 | 65 | -10.09 | 6.28E-24 | 3.03E-21 |
| TOM1L2 | 17 | 0.0616 | rs4925108 | 10.35 | rs7501812 | 0.11 | -6.9 | 8.6667 | 117 | 12 | -9.127 | 7.03E-20 | 2.55E-17 |
| IFT122 | 3 | 0.0336 | rs9837325 | -15.41 | rs6439170 | NA | 0 | -5.111 | 90 | 90 | -8.915 | 4.89E-19 | 1.42E-16 |
| B3GAT3 | 11 | 0.0506 | rs11231144 | 10.53 | rs644616 | 0.04035 | -4.78 | 7.69 | 51 | 7 | -8.265 | 1.40E-16 | 3.38E-14 |
| CAND2 | 3 | 0.4266 | rs11718898 | 8.26 | rs13061128 | NA | 0 | -0.346 | 95 | 6 | -7.856 | 3.96E-15 | 8.20E-13 |
| TKT | 3 | 0.1114 | rs11130357 | 10.25 | rs3736151 | 0.09241 | 6.22 | 7.682 | 93 | 21 | 7.646 | 2.07E-14 | 3.75E-12 |
| MKRN2 | 3 | 0.1604 | rs5746255 | -8.9 | rs11710163 | 0.08302 | 5.99 | -7.485 | 84 | 21 | -7.438 | 1.02E-13 | 1.64E-11 |
| DNM3 | 1 | 0.1005 | rs714515 | 16.24 | rs7528296 | 0.04173 | -5.38 | -10.1667 | 389 | 389 | 7.346 | 2.04E-13 | 2.96E-11 |
| PLXND1 | 3 | 0.1061 | rs9837325 | -15.41 | rs746210 | 0.00263 | -3.07 | -11.903 | 73 | 73 | 7.318 | 2.51E-13 | 3.08E-11 |
| PLCB3 | 11 | 0.033 | rs35169799 | 9.5 | rs11603192 | 0.00062 | 3.43 | 2.3158 | 81 | 6 | 7.316 | 2.55E-13 | 3.08E-11 |
| PDE4C | 19 | 0.0914 | rs12608504 | 14.33 | rs273506 | NA | 0 | 5 | 133 | 6 | -7.172 | 7.39E-13 | 8.24E-11 |
| NEK4 | 3 | 0.0616 | rs1108842 | 12.41 | rs13083798 | NA | 0 | 12.294 | 112 | 112 | 7.057 | 1.71E-12 | 1.77E-10 |
| PDDC1 | 11 | 0.0416 | rs10902223 | 8.31 | rs4073591 | NA | 0 | -2.2583 | 104 | 14 | -7.029 | 2.08E-12 | 2.01E-10 |
| SHMT1 | 17 | 0.2789 | rs2746025 | -7.74 | rs2461838 | 0.315 | 10.95 | -7.35 | 58 | 4 | -6.742 | 1.57E-11 | 1.42E-09 |
| ANXA5 | 4 | 0.2108 | rs7680787 | -7.67 | rs13145977 | 0.24681 | -10.06 | -6.45 | 124 | 3 | 6.67 | 2.56E-11 | 2.18E-09 |
| AAGAB | 15 | 0.0346 | rs7166081 | -6.7 | rs17213990 | NA | 0 | 4.2963 | 120 | 120 | 6.571 | 4.99E-11 | 4.02E-09 |
| NDUFS1 | 2 | 0.0206 | rs3732083 | 6.33 | rs818018 | 0.02175 | 3.6 | -4.7778 | 91 | 7 | -6.52 | 7.04E-11 | 5.37E-09 |
| CTSB | 8 | 0.0918 | rs2409836 | 7.47 | rs1293322 | 0.08741 | 6.44 | 6.5 | 145 | 145 | 6.474 | 9.55E-11 | 6.92E-09 |
| TPPP3 | 16 | 0.0586 | rs8055190 | -7.34 | rs3868143 | 0.05622 | -5.01 | -6.1613 | 50 | 3 | 6.379 | 1.78E-10 | 1.23E-08 |
| DISP2 | 15 | 0.0743 | rs2289328 | 6.7 | rs8037267 | NA | 0 | 0.7879 | 99 | 1 | 6.333 | 2.40E-10 | 1.58E-08 |
| NRP1 | 10 | 0.1089 | rs734187 | -5.9 | rs2755979 | NA | 0 | -0.1111 | 225 | 225 | 6.048 | 1.46E-09 | 9.20E-08 |
| EHD4 | 15 | 0.0569 | rs2303518 | 7.95 | rs11549015 | 0.03228 | -4.66 | 5.5833 | 174 | 8 | -5.692 | 1.25E-08 | 7.55E-07 |
| TBC1D13 | 9 | 0.1189 | rs10988134 | 5.95 | rs15676 | 0.08056 | 6.21 | 5.7895 | 52 | 20 | 5.684 | 1.31E-08 | 7.59E-07 |
| ENDOG | 9 | 0.0462 | rs10988134 | 5.95 | rs3750325 | NA | 0 | -0.6512 | 58 | 5 | -5.638 | 1.72E-08 | 9.59E-07 |
| LLGL1 | 17 | 0.0516 | rs12939469 | -9.22 | rs4925159 | 0.0439 | 4.41 | -4.7647 | 101 | 101 | -5.562 | 2.66E-08 | 1.39E-06 |
| NSF | 17 | 0.1746 | rs199501 | 7.25 | rs7226263 | ####### | 4.65 | 0.8605 | 54 | 54 | 5.561 | 2.68E-08 | 1.39E-06 |
| ECHDC1 | 6 | 0.0569 | rs9968920 | -22.35 | rs3823043 | 0.00164 | 3.26 | 2.25 | 65 | 65 | 5.539 | 3.05E-08 | 1.52E-06 |
| HINT3 | 6 | 0.0232 | rs9398794 | 7.35 | rs2024583 | 0.01628 | -3.64 | -5.4737 | 61 | 61 | 5.374 | 7.70E-08 | 3.72E-06 |
| SLC44A2 | 19 | 0.0264 | rs11085744 | -6.06 | rs11085744 | 0.01507 | 4.07 | -6.0556 | 85 | 85 | -5.367 | 8.01E-08 | 3.74E-06 |
| PTPMT1 | 11 | 0.0511 | rs4752783 | 4.92 | rs1044269 | NA | 0 | 4.2174 | 38 | 6 | 5.284 | 1.26E-07 | 5.71E-06 |
| AUH | 9 | 0.0917 | rs16907277 | 7.86 | rs4743820 | 0.0872 | 6.94 | 2.2105 | 130 | 8 | 5.266 | 1.39E-07 | 6.05E-06 |
| ATP13A2 | 1 | 0.0567 | rs9435732 | -6.74 | rs2076606 | NA | 0 | 0.8925 | 113 | 113 | 5.263 | 1.42E-07 | 6.05E-06 |
| GANAB | 11 | 0.2081 | rs11231144 | 10.53 | rs1058678 | 0.05436 | -4.98 | -7.3333 | 56 | 56 | 5.205 | 1.94E-07 | 8.03E-06 |
| NENF | 1 | 0.0421 | rs11590242 | 6.27 | rs11119918 | NA | 0 | -1.2308 | 104 | 4 | 5.163 | 2.44E-07 | 9.82E-06 |
| CARM1 | 19 | 0.0305 | rs8106691 | -5.52 | rs11085749 | 0.02938 | 4.46 | -4.2222 | 70 | 5 | -5.133 | 2.86E-07 | 1.12E-05 |
| HTT | 4 | 0.0347 | rs362275 | -5.84 | rs2857839 | -0.0026 | 3.42 | -4.7778 | 138 | 138 | -5.095 | 3.48E-07 | 1.33E-05 |
| C3orf18 | 3 | 0.0602 | rs34166957 | 5.66 | rs2236984 | NA | 0 | 4.28 | 49 | 8 | 4.991 | 6.00E-07 | 2.23E-05 |
| SNAP47 | 1 | 0.079 | rs17555357 | -5.72 | rs6703846 | 0.02609 | 4.65 | 4.3158 | 92 | 92 | 4.984 | 6.23E-07 | 2.26E-05 |
| CORO7 | 16 | 0.0833 | rs886860 | 10.96 | rs6500596 | 0.06139 | -5 | 7.0952 | 83 | 4 | -4.969 | 6.75E-07 | 2.34E-05 |
| RMDN3 | 15 | 0.1785 | rs2412546 | -6 | rs1142468 | 0.0848 | -7.21 | -2.8576 | 53 | 9 | 4.968 | 6.78E-07 | 2.34E-05 |
| GMPPB | 3 | 0.1648 | rs2291542 | 6.89 | rs6809879 | 0.17723 | 8.21 | -4.087 | 79 | 6 | -4.949 | 7.48E-07 | 2.52E-05 |
| FLNC | 7 | 0.0407 | rs339054 | -4.39 | rs9656375 | 0.00017 | 3.61 | -1.8571 | 96 | 96 | -4.902 | 9.49E-07 | 3.13E-05 |
| RFT1 | 3 | 0.0815 | rs11130357 | 10.16 | rs2115779 | NA | 0 | 1.158 | 88 | 88 | -4.889 | 1.02E-06 | 3.28E-05 |
| PML | 15 | 0.0648 | rs2165241 | -7.11 | rs9944214 | -0.0027 | -3.21 | 3.649 | 84 | 84 | -4.866 | 1.14E-06 | 3.51E-05 |
| MFI2 | 3 | 0.1024 | rs3843376 | 5.17 | rs6583187 | NA | 0 | 1.905 | 103 | 15 | -4.866 | 1.14E-06 | 3.51E-05 |
| MOXD1 | 6 | 0.0565 | rs3757298 | 6.83 | rs4897569 | NA | 0 | -1.2778 | 133 | 133 | -4.858 | 1.19E-06 | 3.59E-05 |
| JMJD7 | 15 | 0.0748 | rs2303518 | 7.95 | rs1918314 | NA | 0 | -2.2273 | 96 | 2 | 4.724 | 2.31E-06 | 6.83E-05 |
| DUS2 | 16 | 0.0606 | rs5923 | -6.54 | rs7187289 | NA | 0 | -3.7727 | 48 | 48 | -4.689 | 2.75E-06 | 7.97E-05 |
| HIP1R | 12 | 0.134 | rs11060406 | 9.67 | rs11060180 | 0.05238 | 4.6 | 3.4737 | 65 | 3 | 4.674 | 2.95E-06 | 8.36E-05 |
| ATP13A1 | 19 | 0.0932 | rs2304128 | 5.56 | rs2304130 | 0.08058 | 5.93 | 4.9032 | 94 | 3 | 4.671 | 3.00E-06 | 8.36E-05 |
| SUSD5 | 3 | 0.2114 | rs4678756 | -4.26 | rs12632196 | NA | 0 | 1.741 | 106 | 6 | 4.658 | 3.20E-06 | 8.75E-05 |
| ACOT7 | 1 | 0.066 | rs6692407 | 5.23 | rs3789498 | 0.08316 | -6.47 | 4.3264 | 114 | 5 | -4.627 | 3.71E-06 | 9.96E-05 |
| TMCC2 | 1 | 0.0499 | rs3851294 | -8.29 | rs1180734 | 0.04193 | -4.96 | -3.1053 | 114 | 7 | 4.569 | 4.90E-06 | 0.000129 |
| DCXR | 17 | 0.0637 | rs34600945 | 6.63 | rs34319293 | NA | 0 | 5.8698 | 61 | 61 | -4.509 | 6.52E-06 | 0.000169 |
| CAPN2 | 1 | 0.0591 | rs6604731 | -5.45 | rs6678103 | 0.03378 | -4.03 | -5.2857 | 117 | 117 | 4.472 | 7.76E-06 | 0.000197 |
| PNKD | 2 | 0.0298 | rs10193189 | 5.98 | rs10175470 | NA | 0 | -0.7306 | 118 | 7 | 4.464 | 8.04E-06 | 0.000201 |
| NUCB2 | 11 | 0.0907 | rs7928810 | 6.28 | rs739689 | 0.02879 | 4.85 | -2.8089 | 97 | 97 | -4.427 | 9.56E-06 | 0.000231 |
| NRBF2 | 10 | 0.0363 | rs7073746 | -5.06 | rs10995404 | NA | 0 | -1.7857 | 77 | 2 | 4.426 | 9.58E-06 | 0.000231 |
| SEC14L2 | 22 | 0.0939 | rs13058399 | 7.333 | rs5753103 | 0.01412 | -3.98 | -0.9412 | 130 | 130 | 4.422 | 9.80E-06 | 0.000232 |
| LACTB2 | 8 | 0.0855 | rs13280922 | 6.39 | rs12681420 | 0.08932 | 6.6 | -2.7222 | 66 | 7 | -4.418 | 9.97E-06 | 0.000232 |
| EFTUD1 | 15 | 0.1182 | rs2654210 | 4.67 | rs2654206 | 0.01858 | 5.38 | 3.8947 | 128 | 128 | 4.414 | 1.01E-05 | 0.000232 |
| NME1-NME2 | 17 | 0.0882 | rs9896627 | -5.05 | rs10514981 | 0.0635 | -5.1 | -3.9091 | 67 | 3 | 4.404 | 1.06E-05 | 0.000239 |
| TYW5 | 2 | 0.0451 | rs1509833 | 4.89 | rs769957 | NA | 0 | -3.2632 | 75 | 3 | 4.403 | 1.07E-05 | 0.000239 |
| TREX1 | 3 | 0.0435 | rs2267846 | -9.95 | rs4558783 | NA | 0 | 2.368 | 62 | 3 | -4.371 | 1.24E-05 | 0.000272 |
| PDIA4 | 7 | 0.1474 | rs2709331 | -4.5 | rs12534095 | 0.18448 | 8.59 | -3.6818 | 64 | 8 | -4.364 | 1.28E-05 | 0.000277 |
| CSPG4 | 15 | 0.0292 | rs4886722 | -4.3 | rs4503758 | -0.0025 | -3.31 | 3.8947 | 60 | 60 | -4.342 | 1.41E-05 | 0.0003 |
| FAM162B | 6 | 0.1411 | rs654128 | -3.8 | rs6923075 | NA | 0 | 3.15 | 51 | 51 | 4.323 | 1.54E-05 | 0.000323 |
| MECR | 1 | 0.24 | rs547976 | -5.58 | rs547976 | 0.07892 | 5.91 | -5.5789 | 101 | 41 | -4.311 | 1.63E-05 | 0.000337 |
| NCOA7 | 6 | 0.1149 | rs6918302 | -6.45 | rs10872303 | 0.00761 | 4.15 | -5.7222 | 109 | 109 | -4.276 | 1.90E-05 | 0.000388 |
| DLD | 7 | 0.032 | rs11766345 | -9.97 | rs2237690 | 0.01152 | 4.5 | -3.9444 | 146 | 3 | -4.254 | 2.10E-05 | 0.000423 |
| CYBRD1 | 2 | 0.2201 | rs17221848 | 6.57 | rs7590275 | NA | 0 | 2.1053 | 113 | 15 | -4.244 | 2.20E-05 | 0.000433 |
| EML2 | 19 | 0.0732 | rs12978792 | 5.82 | rs7252175 | 0.09066 | -6.08 | 4.037 | 86 | 4 | -4.243 | 2.21E-05 | 0.000433 |
| PAFAH1B2 | 11 | 0.0489 | rs4938353 | 4.36 | rs4938354 | 0.01204 | -3.62 | 3.4762 | 85 | 85 | -4.223 | 2.41E-05 | 0.000466 |
| CCBL2 | 1 | 0.3634 | rs10801687 | -5.11 | rs2765527 | 0.46717 | -13.55 | -3.7778 | 93 | 10 | 4.219 | 2.45E-05 | 0.000467 |
| LRRC8A | 9 | 0.0667 | rs10988134 | 5.95 | rs10988151 | 0.04451 | -4.77 | 3.7368 | 73 | 4 | -4.216 | 2.48E-05 | 0.000467 |
| DHRS11 | 17 | 0.1559 | rs2306589 | 5.53 | rs35712149 | 0.28 | -10.45 | -4.268 | 101 | 18 | 4.143 | 3.42E-05 | 0.000635 |
| LYSMD2 | 15 | 0.0967 | rs16964505 | -5.33 | rs16964505 | 0.04431 | 4.4 | -5.3256 | 100 | 100 | -4.131 | 3.62E-05 | 0.000664 |
| LUZP1 | 1 | 0.0582 | rs10917360 | 7.4 | rs683893 | 0.01678 | 3.94 | 2.8856 | 97 | 97 | 4.111 | 3.94E-05 | 0.000714 |
| ASAP2 | 2 | 0.0383 | rs6745500 | -5.89 | rs11676012 | 0.00246 | -4 | 3.4737 | 159 | 159 | -4.077 | 4.56E-05 | 0.000816 |
| SNUPN | 15 | 0.0614 | rs7163907 | -4.65 | rs7164541 | NA | 0 | 0.6606 | 68 | 68 | -4.045 | 5.23E-05 | 0.000924 |
| SWAP70 | 11 | 0.097 | rs1996794 | -4.62 | rs737617 | 0.05576 | -6.07 | -2 | 122 | 122 | 3.983 | 6.81E-05 | 0.001189 |
| NUDT6 | 4 | 0.2686 | rs1458758 | -8.55 | rs10005301 | NA | 0 | -5.5648 | 89 | 8 | 3.957 | 7.60E-05 | 0.001311 |
| GIMAP4 | 7 | 0.1484 | rs3807382 | 5.38 | rs12531207 | 0.14275 | 7.42 | 3.5 | 114 | 5 | 3.952 | 7.75E-05 | 0.001321 |
| LRRC16A | 6 | 0.1343 | rs9295661 | -6.69 | rs1076668 | 0.02656 | 5.39 | -4.3636 | 332 | 20 | -3.945 | 7.98E-05 | 0.001345 |
| ARL3 | 10 | 0.1608 | rs10883759 | 6.11 | rs2298278 | 0.01235 | -4.39 | -3.4333 | 100 | 100 | 3.935 | 8.33E-05 | 0.001387 |
| MGMT | 10 | 0.1653 | rs10764881 | -4.75 | rs1711673 | NA | 0 | 1.2544 | 286 | 18 | 3.917 | 8.95E-05 | 0.001474 |
| LRPPRC | 2 | 0.1059 | rs4953032 | -3.76 | rs7568481 | 0.05651 | 5.72 | -3.1579 | 177 | 14 | -3.912 | 9.16E-05 | 0.001491 |
| RAB24 | 5 | 0.0583 | rs3088050 | 8.81 | rs28362590 | 0.0528 | 4.97 | -3.7238 | 73 | 2 | -3.895 | 9.82E-05 | 0.001581 |
| SUGP1 | 19 | 0.065 | rs10401969 | 5.79 | rs539 | NA | 0 | 1.5556 | 83 | 12 | -3.886 | 0.000102 | 0.001624 |
| CPNE8 | 12 | 0.0454 | rs2892410 | -4.78 | rs9325144 | 0.00396 | -4.08 | 3.3684 | 143 | 143 | -3.868 | 0.00011 | 0.001733 |
| CAMKK2 | 12 | 0.1432 | rs208296 | -4.78 | rs3794207 | 0.02505 | 4.03 | -2.5263 | 125 | 34 | -3.86 | 0.000113 | 0.001761 |
| PITPNC1 | 17 | 0.0725 | rs12450700 | -7.28 | rs12601587 | 0.00778 | -3.74 | -5.0655 | 193 | 193 | 3.852 | 0.000117 | 0.001804 |
| RWDD1 | 6 | 0.0383 | rs4946182 | 3.96 | rs11961321 | 0.04085 | -5.1 | 3.7619 | 74 | 4 | -3.847 | 0.000119 | 0.001815 |
| PLCL1 | 2 | 0.0682 | rs4850808 | 3.83 | rs1595823 | 0.06886 | 5.79 | 3.3333 | 180 | 10 | 3.834 | 0.000126 | 0.001902 |
| SPAG7 | 17 | 0.1162 | rs16942615 | -3.89 | rs8078173 | NA | 0 | 1.7419 | 92 | 2 | -3.831 | 0.000128 | 0.001912 |
| RABEPK | 9 | 0.0798 | rs13299973 | 5.67 | rs10760392 | NA | 0 | -4.1667 | 65 | 11 | 3.827 | 0.00013 | 0.001922 |
| UROS | 10 | 0.1313 | rs12049644 | -4.5 | rs10794025 | 0.20603 | 9.5 | -4 | 67 | 8 | -3.796 | 0.000147 | 0.002152 |
| NLRX1 | 11 | 0.1256 | rs3825061 | 4.61 | rs4938637 | 0.05939 | 5.25 | 4.5 | 85 | 6 | 3.791 | 0.00015 | 0.002174 |
| MTHFR | 1 | 0.4637 | rs13306560 | -4.46 | rs1801133 | 0.1683 | -8.18 | 3.2222 | 144 | 144 | -3.716 | 0.000203 | 0.002912 |
| AMPD3 | 11 | 0.1611 | rs1349326 | -6.17 | rs11604833 | 0.12333 | 6.91 | -2.9623 | 171 | 171 | -3.713 | 0.000205 | 0.002912 |
| RPAP1 | 15 | 0.057 | rs17676742 | 7.47 | rs35826725 | NA | 0 | 0.3426 | 79 | 79 | -3.705 | 0.000211 | 0.002968 |
| AP3D1 | 19 | 0.0502 | rs12459350 | 8.29 | rs2072304 | 0.0278 | -4.07 | 3.6957 | 72 | 1 | -3.696 | 0.000219 | 0.003036 |
| DGKQ | 4 | 0.1198 | rs4690220 | -9.53 | rs4583705 | 0.11785 | -6.76 | 6.5862 | 110 | 110 | -3.695 | 0.00022 | 0.003036 |
| DEPTOR | 8 | 0.1062 | rs4871827 | 3.79 | rs7833407 | NA | 0 | 0.4571 | 178 | 5 | 3.689 | 0.000225 | 0.003076 |
| FLAD1 | 1 | 0.0322 | rs905938 | -12.15 | rs869506 | NA | 0 | 1.1111 | 74 | 74 | -3.673 | 0.00024 | 0.003247 |
| MAP1S | 19 | 0.109 | rs12979056 | -4 | rs12979056 | 0.13649 | -7.4 | -4 | 91 | 11 | 3.671 | 0.000242 | 0.003247 |
| HARS2 | 5 | 0.0315 | rs753280 | 4.24 | rs778596 | 0.02177 | 3.54 | 3.5556 | 77 | 77 | 3.656 | 0.000256 | 0.003403 |
| FAM134A | 2 | 0.0439 | rs13419740 | -4.83 | rs17655123 | 0.0308 | -4.7 | -3.2593 | 87 | 13 | 3.647 | 0.000265 | 0.003485 |
| ADCY3 | 2 | 0.0378 | rs17046742 | -7.44 | rs6719275 | NA | 0 | -0.34 | 121 | 121 | 3.646 | 0.000267 | 0.003485 |
| SLC25A22 | 11 | 0.0971 | rs10902223 | 8.27 | rs7946354 | 0.02035 | -4.24 | 7.2353 | 100 | 100 | -3.638 | 0.000275 | 0.003558 |
| SLC25A12 | 2 | 0.0587 | rs6716901 | 3.93 | rs6738445 | 0.05831 | 5.57 | 3.8421 | 93 | 93 | 3.621 | 0.000293 | 0.003757 |
| C2CD2L | 11 | 0.0791 | rs3825061 | 4.61 | rs4938626 | 0.06954 | -5.64 | 3.6091 | 101 | 2 | -3.608 | 0.000308 | 0.003914 |
| TXNDC5 | 6 | 0.0554 | rs7764128 | -4.18 | rs9505293 | 0.05231 | 4.7 | -4.0357 | 165 | 4 | -3.604 | 0.000314 | 0.003914 |
| FAM160B2 | 8 | 0.0335 | rs1552286 | 5.15 | rs17296501 | NA | 0 | -2.36 | 91 | 3 | 3.603 | 0.000314 | 0.003914 |
| PNMAL2 | 19 | 0.055 | rs2311223 | -3.29 | rs892106 | 0.01712 | -4.71 | -2.1518 | 89 | 89 | 3.602 | 0.000316 | 0.003914 |
| PLCG1 | 20 | 0.0824 | rs1997833 | 7.26 | rs753381 | 0.05506 | 5.81 | -6 | 54 | 54 | -3.591 | 0.000329 | 0.00404 |
| ITGAM | 16 | 0.0213 | rs1052352 | -4.17 | rs4075052 | 0.01957 | -3.65 | -2.9474 | 76 | 76 | 3.583 | 0.00034 | 0.00414 |
| COA1 | 7 | 0.0696 | rs10268054 | -4.17 | rs6952467 | NA | 0 | 1.5 | 106 | 106 | -3.573 | 0.000352 | 0.00425 |
| CALU | 7 | 0.0829 | rs339054 | -4.39 | rs1043550 | 0.1105 | 6.63 | -3.6667 | 110 | 4 | -3.533 | 0.000411 | 0.004922 |
| USP8 | 15 | 0.0319 | rs4775889 | 4.87 | rs4775886 | 0.01102 | -3.7 | 4.375 | 107 | 107 | -3.517 | 0.000436 | 0.005178 |
| TRAP1 | 16 | 0.1971 | rs7190673 | -4.75 | rs2791 | 0.09944 | -7.91 | -2.2444 | 62 | 23 | 3.5 | 0.000465 | 0.005469 |
| SPRYD4 | 12 | 0.1706 | rs7305370 | 4.65 | rs1043011 | 0.12839 | 7.13 | 3.4348 | 64 | 8 | 3.499 | 0.000468 | 0.005469 |
| C1orf123 | 1 | 0.0765 | rs5174 | 4.33 | rs2297656 | 0.09236 | -6.46 | 3.4211 | 109 | 2 | -3.461 | 0.000538 | 0.006236 |
| PLD1 | 3 | 0.0472 | rs13321742 | 4.79 | rs9844361 | NA | 0 | 0.952 | 191 | 191 | 3.441 | 0.00058 | 0.00667 |
| WBP2 | 17 | 0.0364 | rs2305913 | -4.78 | rs7370 | 0.0235 | -4.77 | -3.55 | 59 | 6 | 3.438 | 0.000586 | 0.006686 |
| PPCDC | 15 | 0.0602 | rs8042694 | -3.26 | rs11854704 | NA | 0 | -2.1053 | 67 | 67 | -3.432 | 6.00E-04 | 0.006751 |
| SYNM | 15 | 0.0777 | rs2602026 | 3.9 | rs1703770 | 0.05186 | -5.47 | -2.5238 | 162 | 3 | 3.431 | 0.000601 | 0.006751 |
| TTC19 | 17 | 0.0322 | rs2301652 | 4.67 | rs758853 | 0.0281 | -3.88 | 3.8889 | 73 | 5 | -3.424 | 0.000617 | 0.006877 |
| FSCN1 | 7 | 0.038 | rs852392 | 4.68 | rs4320462 | 0.00962 | 3.45 | 3.6667 | 76 | 76 | 3.397 | 0.000682 | 0.007506 |
| PGS1 | 17 | 0.1001 | rs2376585 | -7.61 | rs7212707 | NA | 0 | -0.0333 | 140 | 6 | 3.394 | 0.000688 | 0.007506 |
| PSMB3 | 17 | 0.5762 | rs764190 | 4.88 | rs764190 | 0.173 | -8.32 | 4.8823 | 74 | 24 | -3.394 | 0.000689 | 0.007506 |
| RGS6 | 14 | 0.1183 | rs17103830 | 3.81 | rs36341 | 0.06614 | -5.56 | -1.4444 | 550 | 550 | 3.375 | 0.000738 | 0.00798 |
| ACP1 | 2 | 0.2398 | rs11693990 | -4.11 | rs9213 | 0.35606 | -11.7 | 3.3684 | 96 | 6 | -3.369 | 0.000756 | 0.008114 |
| CENPV | 17 | 0.0329 | rs11650427 | -3.5 | rs17779879 | 0.0646 | 5.08 | 2.9231 | 73 | 3 | 3.355 | 0.000793 | 0.008449 |
| EMB | 5 | 0.0857 | rs7729211 | 3.64 | rs7448495 | NA | 0 | 2.1053 | 53 | 53 | 3.338 | 0.000844 | 0.008927 |
| TM7SF2 | 11 | 0.0699 | rs10501396 | -4.18 | rs17146575 | NA | 0 | 0.4977 | 87 | 87 | -3.335 | 0.000852 | 0.008946 |
| FOXRED1 | 11 | 0.036 | rs580145 | -3.72 | rs8177348 | 0.0466 | -4.68 | -3.3308 | 139 | 1 | 3.331 | 0.000866 | 0.008994 |
| GOPC | 6 | 0.1322 | rs9489217 | 3.99 | rs1472591 | 0.12633 | 7.71 | 2.85 | 99 | 14 | 3.33 | 0.000869 | 0.008994 |
| RNH1 | 11 | 0.1542 | rs7944548 | -3.85 | rs11823647 | 0.21498 | -9.19 | -3.3684 | 79 | 5 | 3.325 | 0.000885 | 0.009095 |
| DPYSL5 | 2 | 0.1548 | rs12992046 | 4.68 | rs41448746 | 0.07968 | -6.38 | -2.7292 | 87 | 87 | 3.284 | 0.00102 | 0.010408 |
| CDH13 | 16 | 0.2503 | rs9933518 | -3.47 | rs9933518 | 0.05493 | 5.58 | -3.4737 | 1385 | 1385 | -3.28 | 0.00104 | 0.010538 |
| HLCS | 21 | 0.0882 | rs2256034 | 4 | rs2845778 | NA | 0 | -0.556 | 193 | 193 | 3.277 | 0.00105 | 0.010566 |
| AAMDC | 11 | 0.2678 | rs4944178 | 3.38 | rs3819211 | 0.37575 | -12.16 | 3.2414 | 85 | 29 | -3.275 | 0.00106 | 0.010593 |
| EFNA3 | 1 | 0.047 | rs905938 | -12.15 | rs10908449 | NA | 0 | -4.8261 | 88 | 88 | -3.264 | 0.0011 | 0.010917 |
| TRIM47 | 17 | 0.0673 | rs10852766 | -5 | rs1060120 | NA | 0 | 1.5556 | 62 | 6 | 3.252 | 0.00115 | 0.011336 |
| DBR1 | 3 | 0.0263 | rs2246945 | 3.68 | rs329389 | NA | 0 | 0.148 | 64 | 64 | 3.249 | 0.00116 | 0.011357 |
| PRKCD | 3 | 0.1064 | rs11130357 | 10.18 | rs750527 | 0.05692 | -5.17 | 5.61 | 106 | 6 | -3.237 | 0.00121 | 0.011767 |
| SH3PXD2B | 5 | 0.1601 | rs6866204 | 6.16 | rs2731688 | 0.06745 | 5.98 | -4.4737 | 161 | 38 | -3.235 | 0.00122 | 0.011785 |
| ARHGAP21 | 10 | 0.0491 | rs7087801 | 4.75 | rs7911992 | 0.01262 | -3.63 | -1.2632 | 139 | 139 | 3.222 | 0.00127 | 0.012187 |
| IDH3A | 15 | 0.1275 | rs11072729 | -3.75 | rs3816253 | 0.16832 | 8.34 | 2.6667 | 119 | 23 | 3.202 | 0.00137 | 0.01306 |
| ECI1 | 16 | 0.3672 | rs12921326 | -4.27 | rs11645942 | 0.32862 | -11.16 | -3.1526 | 60 | 3 | 3.168 | 0.00153 | 0.01449 |
| RPE | 2 | 0.0575 | rs1861072 | -3.39 | rs2723211 | 0.06888 | 5.86 | -3.2222 | 46 | 4 | -3.152 | 0.00162 | 0.015144 |
| CHL1 | 3 | 0.1727 | rs17050452 | -4.37 | rs3956142 | 0.02386 | 5.7 | 1.571 | 246 | 15 | 3.152 | 0.00162 | 0.015144 |
| GBAS | 7 | 0.1409 | rs7793921 | 2.85 | rs7793921 | 0.12411 | 7.01 | 2.85 | 85 | 4 | 3.148 | 0.00164 | 0.015233 |
| OLFM2 | 19 | 0.0425 | rs12459673 | 6.86 | rs12979274 | 0.02915 | 4.6 | 3 | 85 | 85 | 3.145 | 0.00166 | 0.015321 |
| CHKB-CPT1B | 22 | 0.0976 | rs6010044 | 3.682 | rs17001608 | -0.0023 | 3.02 | -0.5405 | 104 | 104 | -3.142 | 0.00168 | 0.015407 |
| ABCB9 | 12 | 0.0532 | rs11060406 | 9.67 | rs11060942 | 0.05161 | 4.64 | 7.4818 | 50 | 2 | 3.118 | 0.00182 | 0.016586 |
| ACSF2 | 17 | 0.0563 | rs1380657 | -3.8 | rs736911 | 0.0484 | -4.45 | -3.1111 | 113 | 1 | 3.111 | 0.00186 | 0.016845 |
| TSTD1 | 1 | 0.1593 | rs4656985 | 4.22 | rs11265548 | 0.06696 | 6.57 | 2.3793 | 88 | 88 | 3.1 | 0.00194 | 0.017246 |
| TTLL12 | 22 | 0.2783 | rs5759197 | -5.316 | rs47340 | 0.09551 | 7.29 | 2.5789 | 164 | 27 | 3.1 | 0.00194 | 0.017246 |
| CAPG | 2 | 0.1761 | rs7428 | -3.22 | rs3770102 | 0.11122 | 6.75 | 1.9145 | 88 | 88 | 3.099 | 0.00194 | 0.017246 |
| CARS2 | 13 | 0.2787 | rs7337089 | 2.88 | rs7337089 | 0.13196 | -8.25 | 2.875 | 141 | 24 | -3.084 | 0.00204 | 0.018024 |
| CSAD | 12 | 0.0376 | rs12816417 | -4.81 | rs2280446 | NA | 0 | -4.4167 | 85 | 85 | 3.057 | 0.00224 | 0.019671 |
| NT5C | 17 | 0.1047 | rs11657106 | -5.95 | rs729405 | 0.00412 | 3.9 | -3.45 | 66 | 66 | -3.051 | 0.00228 | 0.019902 |
| EIF2B5 | 3 | 0.0302 | rs7622660 | -4.43 | rs843369 | 0.00945 | -3.82 | -3.632 | 93 | 93 | 3.047 | 0.00231 | 0.019924 |
| GBA2 | 9 | 0.1846 | rs2065039 | -4.5 | rs3750434 | 0.20854 | -8.94 | 3.0556 | 104 | 2 | -3.047 | 0.00231 | 0.019924 |
| MPI | 15 | 0.1063 | rs12442901 | -4.26 | rs8031937 | 0.15727 | -8.4 | -3.1053 | 79 | 10 | 3.045 | 0.00233 | 0.019977 |
| AGPAT9 | 4 | 0.0674 | rs11099608 | 3.5 | rs6845316 | NA | 0 | 1.5625 | 108 | 108 | 3.039 | 0.00238 | 0.020286 |
| GSTP1 | 11 | 0.1171 | rs3758938 | -5.3 | rs7941648 | 0.18313 | -8.99 | -2.2222 | 76 | 4 | 3.021 | 0.00252 | 0.02123 |
| EIF2B3 | 1 | 0.0393 | rs767975 | -5.17 | rs11573562 | 0.01148 | 4.51 | 1.9203 | 102 | 102 | 3.02 | 0.00252 | 0.02123 |
| NT5C3B | 17 | 0.1682 | rs9907244 | 5.95 | rs7212972 | 0.173 | 8.16 | -3.2776 | 94 | 12 | -3.01 | 0.00261 | 0.021818 |
| TXNL4A | 18 | 0.0313 | rs891488 | -3.3 | rs3809929 | NA | 0 | -1.391 | 109 | 4 | -3.009 | 0.00262 | 0.021818 |
| NANS | 9 | 0.2213 | rs3780471 | 3.36 | rs10818468 | 0.23021 | -9.47 | -2.6191 | 109 | 109 | 3.003 | 0.00267 | 0.022108 |
| DLG1 | 3 | 0.1955 | rs1134986 | -5.41 | rs9880331 | 0.03454 | 5.13 | -0.679 | 209 | 209 | 2.993 | 0.00276 | 0.022723 |
| MAN2C1 | 15 | 0.3149 | rs4886707 | -4.2 | rs9673084 | 0.10493 | -6.36 | 3.9 | 30 | 6 | -2.977 | 0.00291 | 0.023823 |
| MICAL3 | 22 | 0.0743 | rs9605473 | -4.238 | rs401910 | 0.00128 | -3.61 | 2.9642 | 270 | 270 | -2.968 | 0.00299 | 0.024285 |
| PLEKHA6 | 1 | 0.0881 | rs3747636 | -6.14 | rs4338423 | -0.0008 | -4.01 | -1.9643 | 239 | 239 | 2.967 | 0.003 | 0.024285 |
| GRK5 | 10 | 0.0607 | rs17608274 | 5.08 | rs7906373 | NA | 0 | -0.2778 | 221 | 221 | -2.96 | 0.00308 | 0.024794 |
| FARSB | 2 | 0.0944 | rs11692810 | -3.21 | rs4674677 | 0.0737 | -6.24 | -2.7895 | 140 | 7 | 2.952 | 0.00316 | 0.025238 |
| TMEM120A | 7 | 0.067 | rs17148944 | -3.45 | rs7223 | NA | 0 | -0.488 | 73 | 8 | 2.951 | 0.00317 | 0.025238 |
| ABHD6 | 3 | 0.0595 | rs4681821 | -5.11 | rs1554128 | 0.00119 | 3.6 | -2.65 | 128 | 128 | -2.945 | 0.00323 | 0.025575 |
| CAMLG | 5 | 0.2973 | rs2108665 | 2.93 | rs11747528 | NA | 0 | -0.3996 | 29 | 4 | -2.926 | 0.00344 | 0.02709 |
| ADSL | 22 | 0.0811 | rs713898 | 2.808 | rs8135371 | 0.08217 | 5.88 | 2.5769 | 64 | 2 | 2.921 | 0.00349 | 0.027335 |
| ASRGL1 | 11 | 0.1719 | rs2509963 | -8.5 | rs2463833 | 0.1917 | 8.79 | 4.3333 | 119 | 7 | 2.917 | 0.00354 | 0.027578 |
| ULK3 | 15 | 0.1771 | rs2168519 | -4.53 | rs936227 | 0.25597 | 9.94 | -2.2778 | 87 | 8 | -2.915 | 0.00356 | 0.027585 |
| CS | 12 | 0.0318 | rs2695789 | 3.31 | rs2306693 | 0.01334 | -3.18 | 2.5714 | 42 | 42 | -2.907 | 0.00365 | 0.028132 |
| NADK2 | 5 | 0.111 | rs10941278 | 4.22 | rs33671 | -0.0027 | -2.66 | -1.2593 | 79 | 79 | -2.905 | 0.00367 | 0.028137 |
| LY6H | 8 | 0.0709 | rs11781387 | -4 | rs10109061 | 0.04843 | -5.4 | -3.5 | 78 | 78 | 2.9 | 0.00373 | 0.028446 |
| FAM120B | 6 | 0.0954 | rs2180052 | -3.56 | rs9348260 | NA | 0 | 1.2105 | 155 | 10 | 2.885 | 0.00391 | 0.029663 |
| ISOC1 | 5 | 0.0428 | rs257936 | 2.86 | rs7735174 | 0.01346 | 3.53 | 2.6111 | 89 | 89 | 2.883 | 0.00394 | 0.029731 |
| PACSIN2 | 22 | 0.1045 | rs1040427 | -3.278 | rs17415203 | 0.0319 | 5.57 | 1.4643 | 163 | 18 | 2.881 | 0.00396 | 0.029731 |
| PFKFB3 | 10 | 0.1119 | rs2274358 | -3.55 | rs10905806 | NA | 0 | -3.2105 | 166 | 166 | 2.876 | 0.00403 | 0.0301 |
| NOSTRIN | 2 | 0.0533 | rs3931 | 3.74 | rs6709565 | NA | 0 | -1.5 | 171 | 171 | -2.874 | 0.00406 | 0.030169 |
| PPOX | 1 | 0.1805 | rs4656994 | 5.59 | rs36013429 | 0.15311 | -7.67 | 2.5569 | 115 | 10 | -2.868 | 0.00413 | 0.030533 |
| GPX1 | 3 | 0.2944 | rs13096474 | -8.53 | rs17080528 | 0.33445 | -11.25 | 2.528 | 52 | 10 | -2.857 | 0.00428 | 0.031481 |
| HHATL | 3 | 0.1097 | rs13061576 | -5.27 | rs4549 | NA | 0 | -1.211 | 95 | 9 | 2.852 | 0.00434 | 0.031761 |
| AMACR | 5 | 0.2572 | rs10074193 | -6.08 | rs11742467 | NA | 0 | 0.3462 | 97 | 8 | -2.845 | 0.00443 | 0.032257 |
| NT5C2 | 10 | 0.0375 | rs17115419 | 4.5 | rs11191506 | 0.02751 | 3.93 | 1.7619 | 95 | 95 | 2.84 | 0.00451 | 0.032675 |
| WDSUB1 | 2 | 0.2889 | rs17494490 | 3.37 | rs2159876 | 0.3475 | -11.81 | -2 | 132 | 12 | 2.831 | 0.00463 | 0.033377 |
| C6orf211 | 6 | 0.0492 | rs4425604 | 3.1 | rs7771156 | 0.05496 | -4.69 | -1.6842 | 97 | 97 | 2.825 | 0.00473 | 0.033905 |
| CRAT | 9 | 0.0233 | rs4836643 | -3.17 | rs7849270 | 0.00265 | -3.42 | -1.7368 | 65 | 65 | 2.824 | 0.00475 | 0.033905 |
| CORO1C | 12 | 0.1602 | rs10746131 | 2.56 | rs1861642 | 0.05432 | -6 | -1.4539 | 129 | 129 | 2.821 | 0.00479 | 0.033998 |
| WIPI1 | 17 | 0.1084 | rs8080306 | -4.1 | rs11657610 | NA | 0 | 0.2778 | 113 | 12 | -2.82 | 0.00481 | 0.033998 |
| SLC9A3R2 | 16 | 0.0959 | rs7185040 | -5.22 | rs7185040 | 0.03115 | -4.47 | -5.2174 | 63 | 21 | 2.816 | 0.00486 | 0.034185 |
| RAB5C | 17 | 0.0336 | rs8082391 | 7.63 | rs8081327 | 0.0288 | 4.16 | 0.7328 | 50 | 50 | 2.804 | 0.00504 | 0.03528 |
| BCS1L | 2 | 0.0403 | rs10178538 | -3.26 | rs12991539 | 0.00487 | -3.18 | -2.023 | 84 | 84 | 2.801 | 0.0051 | 0.035528 |
| ARNT2 | 15 | 0.0603 | rs12908010 | -3.58 | rs3901896 | 0.01431 | 4.49 | -3.4737 | 175 | 175 | -2.796 | 0.00518 | 0.035913 |
| ASPHD1 | 16 | 0.168 | rs11860782 | -4.61 | rs34286592 | NA | 0 | 3.6177 | 67 | 67 | 2.785 | 0.00536 | 0.036809 |
| SGTB | 5 | 0.0923 | rs12517858 | -2.9 | rs1549192 | 0.08505 | -6.08 | -2.7143 | 122 | 3 | 2.785 | 0.00536 | 0.036809 |
| FDXR | 17 | 0.3056 | rs509911 | -2.91 | rs690514 | 0.426 | -12.71 | -2.8636 | 102 | 5 | 2.764 | 0.0057 | 0.038959 |
| LACTB | 15 | 0.154 | rs4774478 | -3.5 | rs2729835 | 0.21167 | -9.06 | -3.4444 | 93 | 12 | 2.758 | 0.00582 | 0.039592 |
| DAAM1 | 14 | 0.1878 | rs11627049 | 2.13 | rs1253035 | 0.02549 | -4.33 | -0.9643 | 166 | 166 | 2.755 | 0.00587 | 0.039746 |
| GIMAP8 | 7 | 0.0951 | rs1608158 | 5.48 | rs6973612 | NA | 0 | -0.5769 | 123 | 11 | -2.74 | 0.00614 | 0.041381 |
| PALM2 | 9 | 0.051 | rs2209815 | -5.67 | rs12684676 | 0.04236 | 4.78 | 1.7826 | 321 | 6 | 2.738 | 0.00617 | 0.04139 |
| IDUA | 4 | 0.1795 | rs4690220 | -9.48 | rs2279181 | NA | 0 | 6.9514 | 110 | 4 | -2.737 | 0.0062 | 0.0414 |
| TRMT11 | 6 | 0.08 | rs9398794 | 7.35 | rs941986 | NA | 0 | -0.1818 | 69 | 69 | 2.729 | 0.00636 | 0.042274 |
| RAB27B | 18 | 0.114 | rs8092503 | -3.05 | rs2871673 | 0.153 | -7.92 | -3 | 93 | 19 | 2.724 | 0.00645 | 0.042482 |
| ECH1 | 19 | 0.0674 | rs9419 | 3.56 | rs2229259 | 0.0865 | -5.86 | 2.2431 | 81 | 3 | -2.724 | 0.00645 | 0.042482 |
| PGAM2 | 7 | 0.1992 | rs17546439 | -4.22 | rs11766570 | NA | 0 | 2.8932 | 62 | 3 | 2.718 | 0.00657 | 0.043077 |
| KIAA1217 | 10 | 0.0863 | rs7082393 | 4.65 | rs11597573 | 0.01298 | -3.7 | 0.2 | 308 | 308 | 2.708 | 0.00677 | 0.04399 |
| ARHGEF10 | 8 | 0.1195 | rs4875950 | 2.88 | rs2957060 | NA | 0 | 0.0526 | 264 | 264 | 2.708 | 0.00677 | 0.04399 |
| COX11 | 17 | 0.0706 | rs12945393 | -4.78 | rs8066213 | NA | 0 | -1.1579 | 106 | 4 | -2.699 | 0.00695 | 0.044958 |
| RASAL1 | 12 | 0.0522 | rs10850127 | 6.5 | rs1902955 | 0.00298 | -2.82 | 1.6316 | 87 | 87 | -2.696 | 0.00701 | 0.045144 |
| PCK2 | 14 | 0.0486 | rs4982856 | -3.63 | rs4982856 | 0.00065 | 3.87 | -3.6265 | 68 | 68 | -2.689 | 0.007172 | 0.045983 |
| NMRAL1 | 16 | 0.4516 | rs3747579 | 10.37 | rs3747575 | NA | 0 | 6.5556 | 68 | 68 | 2.685 | 0.00725 | 0.046053 |
| SLC24A4 | 14 | 0.1457 | rs7160605 | -2.86 | rs11623214 | NA | 0 | 0.5385 | 158 | 158 | 2.683 | 0.007299 | 0.046053 |
| DNAJB1 | 19 | 0.0686 | rs1803768 | -4.62 | rs8104440 | 0.02778 | 4.41 | -2.36 | 75 | 3 | -2.683 | 0.00731 | 0.046053 |
| NIT1 | 1 | 0.5301 | rs4656994 | 5.59 | rs1127525 | 0.25579 | -9.92 | -1.3449 | 93 | 6 | 2.682 | 0.00731 | 0.046053 |
| KIF21B | 1 | 0.2063 | rs3753960 | -3.14 | rs502658 | 0.16628 | 8.31 | 1.7826 | 151 | 14 | 2.679 | 0.00739 | 0.046355 |
| LYRM2 | 6 | 0.2192 | rs715203 | 3.12 | rs2026651 | NA | 0 | 2.5263 | 88 | 2 | -2.674 | 0.00749 | 0.04678 |
| RCN1 | 11 | 0.0676 | rs1033584 | 3.38 | rs224633 | 0.08863 | -5.87 | 2.6 | 117 | 6 | -2.672 | 0.00754 | 0.04689 |
| PCMT1 | 6 | 0.1222 | rs2342764 | -2.68 | rs1112730 | 0.10647 | -6.47 | -2.4444 | 87 | 87 | 2.67 | 0.00759 | 0.047 |
| IVD | 15 | 0.194 | rs2289328 | 6.7 | rs7165012 | 0.15628 | -8.08 | -2.5 | 86 | 11 | 2.665 | 0.00771 | 0.047461 |
| DCUN1D2 | 13 | 0.057 | rs9577281 | 3.26 | rs9549398 | NA | 0 | 0.273 | 123 | 123 | -2.664 | 0.00773 | 0.047461 |
| TGOLN2 | 2 | 0.0536 | rs6547600 | -3.28 | rs4247303 | 0.09149 | -6.21 | -2.5556 | 112 | 6 | 2.662 | 0.00777 | 0.047505 |
| ASAH1 | 8 | 0.0378 | rs7508 | -4.45 | rs2285306 | NA | 0 | 0.4 | 180 | 8 | 2.657 | 0.00788 | 0.047877 |
| NUB1 | 7 | 0.0579 | rs875588 | 3.28 | rs394259 | 0.00694 | 3.82 | -0.2 | 140 | 14 | -2.655 | 0.00792 | 0.047877 |
| TPD52 | 8 | 0.0759 | rs6473202 | 3.32 | rs9643750 | 0.05888 | 4.97 | -2.6111 | 112 | 2 | -2.655 | 0.00793 | 0.047877 |
| PDLIM2 | 8 | 0.2523 | rs1545837 | 3.68 | rs2443502 | NA | 0 | 0.0556 | 126 | 22 | -2.65 | 0.00805 | 0.0484 |
| SNX19 | 11 | 0.0727 | rs10894268 | 2.78 | rs6590507 | NA | 0 | -1.125 | 145 | 145 | -2.645 | 0.00818 | 0.048979 |
| GOT2 | 16 | 0.108 | rs257634 | 2.78 | rs12599931 | 0.10565 | 7.08 | 2.1667 | 109 | 109 | 2.643 | 0.00822 | 0.049016 |
| TMEM163 | 2 | 0.0329 | rs6747870 | 3.96 | rs6430538 | 0.02104 | -3.93 | -0.3333 | 204 | 204 | -2.641 | 0.00828 | 0.049171 |
| HSQ, refers to heritability of the protein using common variants. NSNP, indicates the total number of SNPs within 200kb window (100kb up and downstream) of the gene. NWGT, refers to the total number of SNPs that contribute to the weight of the protein. | | | | | | | | | | | | | |

| Table S8. The TWAS of lacunar stroke integrating the lacunar stroke GWAS (N=7,338) with CMC human brain transcriptome and genetic data (N=452) using FUSION. | | | | | | | | | | | | | |
| --- | --- | --- | --- | --- | --- | --- | --- | --- | --- | --- | --- | --- | --- |
| **Gene** | **CHR** | **HSQ** | **BEST.GWAS.ID** | **BEST.GWAS.Z** | **eQTL.ID** | **eQTL.R^2^** | **eQTL.Z** | **eQTL.GWAS.Z** | **NSNP** | **NWGT** | **TWAS.Z** | **TWAS.P** | **TWAS.FDR.Q** |
| NBEAL1 | 2 | 0.1103 | rs6705330 | -5.28 | rs4510208 | 0.079 | -6.09 | -5.138 | 330 | 26 | 5.44631 | 5.14E-08 | 0.000276 |
| ICA1L | 2 | 0.089 | rs6705330 | -5.28 | rs6705330 | 0.064 | -5.56 | -5.284 | 267 | 1 | 5.284 | 1.26E-07 | 0.000284 |
| ALS2CR8 | 2 | 0.1191 | rs6705330 | -5.28 | rs6705330 | 0.085 | -6.62 | -5.284 | 278 | 11 | 5.24179 | 1.59E-07 | 0.000284 |
| ULK4 | 3 | 0.4902 | rs6796210 | -5.66 | rs1716642 | 0.63 | 16.91 | -5.313 | 654 | 43 | -5.04267 | 4.59E-07 | 0.000616 |
| FAM117B | 2 | 0.1319 | rs6705330 | -5.28 | rs7568438 | 0.088 | -6.57 | -2.885 | 287 | 13 | 4.50181 | 6.74E-06 | 0.007235 |
| CENPQ | 6 | 0.3365 | rs2501966 | -4.28 | rs2501966 | 0.339 | 12.46 | -4.277 | 357 | 8 | -4.15864 | 0.000032 | 0.028624 |
| CCND2 | 12 | 0.1331 | rs12580262 | 3.75 | rs3217925 | 0.069 | 6.02 | 3.622 | 592 | 3 | 4.05051 | 5.11E-05 | 0.039179 |
| HSQ, refers to heritability of the mRNA using common variants. NSNP, indicates the total number of SNPs within 200kb window (100kb up and downstream) of the gene. NWGT, refers to the total number of SNPs that contribute to the weight of the mRNA. | | | | | | | | | | | | | |

| Table S9. The lacunar stroke TWAS verified 1 significant gene. | | | | |  |  |  |
| --- | --- | --- | --- | --- | --- | --- | --- |
| Gene | CHR | eQTL.ID | eQTL.R^2^ | NSNP | MODEL | MODELCV.R^2^ | MODELCV.PV |
| ICA1L | 2 | rs6705330 | 0.063932 | 267 | top1 | 0.064 | 0.00000003 |
| eQTL.R^2^, cross-validation R^2^ of the best eQTL in the locus; MODEL, Best performing model; MODELCV.R^2^, cross-validation R^2^ of the best performing model; MODELCV.PV, cross-validation P-value of the best performing model. | | | | | | | |

| Table S10. SNPs located within 1 Mb of each of the 7 proteins with the lowest p-value for association with lacunar stroke. | | | |
| --- | --- | --- | --- |
| Gene | Chr | SNP with lowest p-value | SNP p-value |
| ICA1L | 2 | rs72934535 | 5.23E−8 |
| CAND2 | 3 | rs9873475 | 6.1E−6 |
| ALDH2 | 12 | rs11066309 | 1.08E−7 |
| MADD | 11 | rs2293576 | 5.96E−10 |
| MRVI1 | 11 | rs73409956 | 4.6E−5 |
| CSPG4 | 15 | rs11634290 | 5.2E−5 |
| PTPN11 | 12 | rs11066309 | 1.08E−7 |

Note: These 7 proteins were significantly associated with lacunar stroke at FDR q<0.05 from the meta-analysis of the discovery and replication PWAS of lacunar stroke association p-values are from the Tayler et al, 2021 lacunar stroke GWAS (N=7,338).
